# *miR‐148a* inhibits early relapsed colorectal cancers and the secretion of VEGF by indirectly targeting HIF‐1α under non‐hypoxia/hypoxia conditions

**DOI:** 10.1111/jcmm.14257

**Published:** 2019-03-04

**Authors:** Hsiang‐Lin Tsai, Zhi‐Feng Miao, Yi‐Ting Chen, Ching‐Wen Huang, Yung‐Sung Yeh, I‐Ping Yang, Jaw‐Yuan Wang

**Affiliations:** ^1^ Division of Colorectal Surgery, Department of Surgery Kaohsiung Medical University Hospital, Kaohsiung Medical University Kaohsiung Taiwan; ^2^ Department of Surgery, Faculty of Medicine, College of Medicine Kaohsiung Medical University Kaohsiung Taiwan; ^3^ Department of Medical Research Kaohsiung Medical University Hospital Kaohsiung Taiwan; ^4^ Department of Pathology Kaohsiung Medical University Hospital, Kaohsiung Medical University Kaohsiung Taiwan; ^5^ Department of Pathology, Faculty of Medicine College of Medicine, Kaohsiung Medical University Kaohsiung Taiwan; ^6^ Graduate Institute of Medicine, College of Medicine, Kaohsiung Medical University Kaohsiung Taiwan; ^7^ Graduate Institute of Clinical Medicine, College of Medicine, Kaohsiung Medical University Kaohsiung Taiwan; ^8^ Division of Trauma and Surgical Critical Care, Department of Surgery Kaohsiung Medical University Hospital, Kaohsiung Medical University Kaohsiung Taiwan; ^9^ Department of Nursing, Shu‐Zen College of Medicine and Management Kaohsiung Taiwan

**Keywords:** colorectal cancers, early relapse, HIF‐1α, *miR‐148a*, non‐hypoxia/hypoxia conditions, VEGF

## Abstract

Vascular endothelial growth factor (VEGF) is correlated with angiogenesis and early relapse of colorectal cancer (CRC). This study investigated the role of *miR‐148a* in the regulation of VEGF/angiogenesis and early relapse of CRC. We established a stable clone with *miR‐148a *expression in HCT116 and HT29 cell lines and created a hypoxic condition by using CoCl_2_ to determine the underlying mechanism of *miR‐148a*. The effects of *miR‐148a* on the phosphoryl‐ERK (pERK)/hypoxia‐inducible factor‐1α (HIF‐1α)/VEGF pathway were evaluated through Western blotting and the inhibitory effect of *miR‐148a* on angiogenesis was demonstrated through a tube formation assay. Sixty‐three CRC tissues (28 early relapse and 35 non‐early relapse) were analysed to assess the relationship between *miR‐148a* and HIF‐1α/VEGF. The protein expression of pERK/HIF‐1α/VEGF in HCT116 and HT29 cells was significantly decreased by *miR‐148a* (all *P* < 0.05). The protein expression of VEGF/HIF‐1α was strongly inversely associated with the expression of *miR‐148a* in the 63 CRC tissue samples (all *P* < 0.05). Tube formation assay demonstrated that *miR‐148a* significantly obliterated angiogenesis. *miR‐148a* suppresses VEGF through down‐regulation of the pERK/HIF‐1α/VEGF pathway and might lead to the inhibition of angiogenesis; *miR‐148a* down‐regulation increased the early relapse rate of CRC. This demonstrates that *miR‐148a* is a potential diagnostic and therapeutic target.

## 1 INTRODUCTION

Colorectal cancer (CRC) is the third most common cancer worldwide and accounts for 10% of all new cancer diagnoses. Unfortunately, 20% of patients diagnosed with CRC have metastatic disease.^1^ The recurrence of CRC is mostly a time‐limited phenomenon; 40%‐50% of recurrence events become apparent within the first year after the initial surgical resection.^2^ In addition, the earlier the relapse occurs, the poorer are the overall survival rates.^3^ The growth and proliferation of metastatic CRC (mCRC) depends essentially on two signalling pathways: the vascular endothelial growth factor (VEGF) and epidermal growth factor receptor (EGFR) pathways. Although substantial progress has been made in the past decades regarding the management of this disease, including surgical treatment, radiotherapy and chemotherapy, patients with advanced CRC continue to receive a poor prognosis and have a high death rate.^4^ Consequently, a better understanding of the molecular mechanisms underlying CRC development and progression is urgently needed. More specifically, oligonucleotide therapies using small interfering RNAs, short hairpin RNAs, RNA aptamers and ribozymes have received considerable attention because they enable the targeted delivery of antitumour drugs without significant toxicity or other systemic side effects.^5^, ^6^, ^7^ In this study, we focused on the use of microRNA (*miRNA*) as a potential therapy for CRC.

In humans, *miR‐148a* with 68 nucleotide sequences is located on chromosome 7p15.2. *miR‐148a* performs the common functions of many miRNA species and is implicated in a series of biological processes including cellular proliferation, apoptosis, metastasis and invasion.^8^ In breast cancer cells, the ERK signalling pathway is the key downstream pathway of hypoxia‐inducible factor‐1α (HIF‐1α) and plays an important role in angiogenesis and cancer development. The down‐regulation of *miR‐148a* expression activates the ERK signalling pathway to increase HIF‐1α and VEGF expression.^9^ Moreover, hypoxia is a potential stimulator of VEGF expression and HIF‐1 may regulate the hypoxic expression of VEGF in colon cancer.^10^ We previously demonstrated an association between *miR‐148a* down‐regulation and early relapse in patients with CRC; this finding indicated that *miR‐148a* is a potential biomarker for identifying high‐risk patients with CRC after curative resection.^11^

Tumour angiogenesis is required for tumour development and growth and HIF‐1α plays a pivotal role in this process.^12^ Vascular endothelial growth factor is a target gene of HIF‐1α. Hypoxia‐inducible factor‐1α regulates VEGF expression at the transcriptional level.^13^ In the present study, we identified *miR‐148a*, which may be related to tumour angiogenesis; identified the signalling pathways that are regulated by *miR‐148a*; and determined the role of *miR‐148a* in the angiogenesis of CRC. Therefore, our findings provide evidence of the role and potential mechanism of *miR‐148a* in regulating CRC angiogenesis and early relapse.

## 2 MATERIALS AND METHODS

### 2.1 Study design

**Figure 1:**
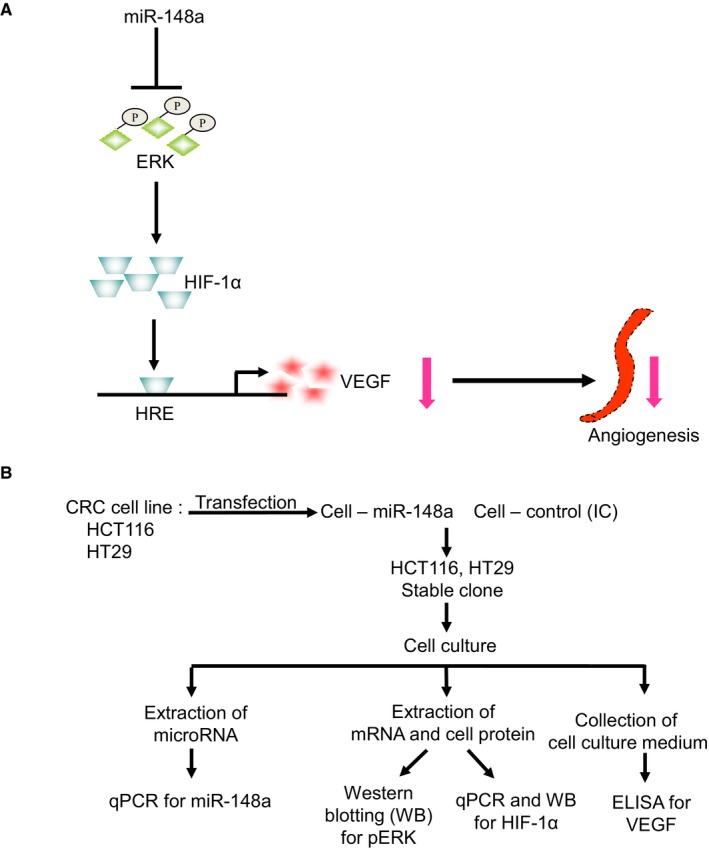
The study hypothesis and design. A, We suggested that that *miR‐148a* inhibits vascular endothelial growth factor (VEGF) through the inactivation of the phosphoryl‐ERK/hypoxia‐inducible factor‐1α (pERK/HIF‐1α) pathway. B, In vitro, we transfected *miR‐148a* into HCT116 and HT29 cells and established stable colorectal cancer (CRC) clones. The protein levels of pERK and HIF‐1α were examined through Western blotting and the mRNA levels of HIF‐1α were tested through RT‐PCR. The protein expression of VEGF was examined through ELISA

In our previous study, we confirmed the relationship between the down‐regulation of *miR‐148a* and post‐operative early relapse.^11^ According to a bioinformatic analysis of pathways,^14^ *miR‐148a *could affect the function of phosphoryl‐ERK [pERK]) and HIF‐1α in other cancers. Hence, we suggested that that *miR‐148a* inhibits VEGF expression by indirectly targeting HIF‐1α and its relevant pathways (Figure 1A). The design of the cell lines study is illustrated in Figure 1B.

### 2.2 Cell lines and cell lines authentication test illustration

Before the selection of cell lines, we tried to use *miR‐148a* for transfection and proliferation in five cell lines: HCT116, HT29, SW480, SW620 and Caco‐2. The results demonstrated that the HCT116 and HT29 cells showed fold changes after transfection and relative proliferation was significant altered (Figure S1A,B) and activation of the *RAS‐RAF *pathway has been reported to be associated with increased VEGF‐induced angiogenesis. For exploring the role of *miR‐148a* in *BRAF *mutation (HT29 cell) and *KRAS* mutation (HCT116 cell), we chose these two cell lines for experimentation. Human CRC cell lines—HCT116 and HT29 cells—were purchased from the Bioresource Collection and Research Center (Hsinchu, Taiwan) and American Type Culture Collection (Manassas, VA) respectively. All cell lines were cultured in DMEM (Gibco, Grand Island, NY) supplemented with 10% foetal bovine serum (Gibco), 100 IU/mL penicillin (Gibco) and 100 µg/mL streptomycin (Gibco) in a humid atmosphere containing 5% CO_2_ at 37°C. Human umbilical vein endothelial cell was a component of Angiogenesis Starter Kit (Thermo Fisher Scientific, Inc, Waltham, MA).

On delivery from provider, the HCT116 and HT29 cell lines were resuscitated and cultured for 2 weeks and then subjected to transfection with pCDH vector (cDNA Cloning and Expression Lentivectors) (System Biosciences, Palo Alto, CA) expressing *miR‐148a*. The transfected cell lines were then used for experiments afterwards. According to the manufacturers manual statement, after transduction in target cells, the pCDH expression construct can integrate into genomic DNA to provide stable, long‐term expression of the target gene. This genomic integration of expression construct would make transfected cell's genome appear different from their wild‐type. Cell line authentication test will no longer, therefore, identify the pCDH transfected cell as original genomic status. As genomic changes were expected, the cell lines used in this study were not subjected to authentication test.

### 2.3 Patient tissue samples

For proving the expression correlation of *miR‐148a* and target protein level, we re‐analysed the expression level of *miR‐148a* in the first cohort of 110 patients^11^ and 63 CRC patients who underwent radical resection were enrolled. Among these patients, 28 were post‐operative early relapse patients with *miR‐148a *non‐overexpression and 35 were post‐operative non‐early relapse patients with *miR‐148a *overexpression. Early relapse was defined as local recurrence (tumor growth restricted to the anastomosis or the region of the primary operation) or distant metastasis (distant metastasis or diffuse peritoneal seeding) within one year after radical resection, and the patients who relapsed after the first year or did not relapse were placed into the non‐early relapse group.^3^, ^11^, ^15^ Written informed consent was obtained from all participants after they had been completely informed of the study protocols and that research was carried out according to the World Medical Association Declaration of Helsinki. This study was approved by the institutional review board of Kaohsiung Medical University Hospital (KMUHIRB‐2012‐03‐02(II)). All methods were performed in accordance with the relevant guidelines and regulations.

### 2.4 Construction of *miR‐148a* overexpressing constructs

A pCDH vector (System Biosciences) was used as an *miR‐148a* overexpression system for assessing the functional consequences of *miR‐148a* overexpression. We constructed the pCDH–*miR‐148a* plasmid by inserting the *miR‐148a* polymerase chain reaction (PCR) product into the multiple cloning sites. The sequences of the primers for *miR‐148a* were GCCTGAATTCATGCTTTTAACGAGTTATTCTTC and CTAGGCGGCCGCGCCTTGCCCCTCCCCCAAGGA. The forward primers were extended at the 5′ end to include the GAATTC sequence and the reverse primers were elongated at their 5′ end to include the GCGGCCGC sequence, which created the *EcoR1* and *Not1* restriction sites respectively. The constructs were confirmed through direct DNA sequencing.

### 2.5 Establishment of a stable clone

The HCT116 and HT29 cells (5 × 10^5^) were seeded and transfected with 400 ng of the constructs (either the negative scrambled pCDH vector or the pCDH–*miR‐148a* plasmid) by using Lipofectamine 2000 (Thermo Fisher Scientific). To select stably transfected HCT116 and HT29 cells containing the pCDH‐negative control or pCDH–*miR‐148a* plasmid, the cells were cultured over 4 weeks in standard culture media supplemented with an additional 12 µg/mL puromycin (Sigma‐Aldrich Inc, St. Louis, MO). Confirmation of stable transfection of the plasmids was obtained using a miRNA real‐time quantitative PCR (RT‐qPCR) assay (Figure S1).

### 2.6 *miR‐148a* expression levels in CRC cell lines

The TaqMan miR RT‐qPCR assay (Applied Biosystems, Foster City, CA) was used to quantify the levels of *miR‐148a*. RT‐qPCR was performed with the Applied Biosystems 7900HT Real‐Time PCR System (Applied Biosystems) according to the default thermal cycling conditions of the ABI 7900 Sequence Detection System 2.4 (Applied Biosystems). The relative expression levels of *miR‐148a* were normalized to that of the internal control U6 snRNA by using the equation log10 (2^−ΔCt^), where ΔCt = Ct*_miR‐148a_* − Ct_U6_.

### 2.7 RNA extraction and cDNA preparation

Approximately 10^7^ cells were harvested from culture plates using trypsin. Total RNA, including mRNAs and miRs, was purified using Qiagen RNAeasy Columns (Qiagen, Hamburg, Germany) according to the manufacturer's protocols. For the miR assay, the cDNA of each miR was synthesized with a unique primer (Applied Biosystems) by using 20 ng of total RNA. For the mRNA quantitative assay, cDNAs were synthesized from 1 μg of total RNA with random hexamer primers by using Reverse Transcriptase (Applied Biosystems).

### 2.8 mRNA expression levels

For the mRNA quantitative assay, RT‐qPCR with SYBR Green (Applied Biosystems) was performed with the Applied Biosystems 7900HT Real‐Time PCR System (Applied Biosystems) according to the default thermal cycling conditions of the ABI 7900 Sequence Detection System 2.4 (Applied Biosystems). The relative expression levels of mRNA were normalized to those of the internal control glyceraldehyde 3‐phosphate dehydrogenase (GAPDH) by using the equation log10 (2^−ΔCt^), where ΔCt = Ct_mRNA_ – Ct_GAPDH_.

### 2.9 Western blotting

Total cell lysates (20 μg) were analysed using sodium dodecyl sulphate–polyacrylamide gel electrophoresis on a 12% gel. After electroblotting onto the nitrocellulose membrane, the membranes were blocked with non‐fat dry milk for 2 hours at room temperature. The membranes were then washed three times with phosphate‐buffered saline (PBS) containing Tween 20 and subsequently incubated with primary antibodies (Abcam plc, Cambridge, England, UK) at 4°C overnight. Anti‐HIF‐1α and anti‐pERK antibody were used at 1:2000 and 1:3000 dilutions respectively. The membranes were then washed with PBS–Tween 20 three times and incubated with a 1:8000 dilution of peroxidase‐linked anti‐mouse IgG (Amersham Biosciences, Piscataway, NJ) for 1 hour at room temperature. After the membranes were washed with PBS‐Tween 20, the bands were detected using a SuperSignal^TM^ West Femto Maximum Sensitivity Substrate (Applied Biosystems).

### 2.10 Enzyme‐linked immunosorbent assay

Vascular endothelial growth factor protein levels in the cell culture medium of CRC cell lines transfected with *miR‐148a* vector were compared with the levels in the medium of control cells. VEGF levels were determined using a VEGF ELISA kit (R&D Systems, Minneapolis, MN) according to the manufacturer's protocol. Briefly, 100 μL of cell culture medium was placed on a microplate that had been pre‐coated with VEGF‐capture antibody at 4°C overnight and the plate was incubated at room temperature for 3 hours. Thereafter, the wells were emptied and rinsed five times with washing buffer. The wells were then incubated with the VEGF detection conjugate. Finally, the plate absorbance was measured at a wavelength of 450 nm.

### 2.11 Immunohistochemistry

Formalin‐fixed, paraffin‐embedded blocks of CRC were collected. All 4‐μm sections were dried, deparaffinized and rehydrated and heat‐mediated antigen retrieval was performed by boiling under pressure in Target Retrieval Buffer (Leica, pH 6.0; Abcam, pH 9.0; and DAKO, pH 9.0 respectively) for 8 minutes. Three percent hydrogen peroxide was also used for 5 minutes to block endogenous peroxidase activity at room temperature and the slides were washed with Tris buffer solution. Immunohistochemistry (IHC) was performed with anti‐HIF‐1α (1:300; Abcam) and anti‐VEGF (1:200; Santa Cruz Biotechnology, Inc, Dallas, TX) as the primary antibodies. Positive and negative control sections were included in each quality control run. The degree of immunostaining was reviewed and scored by two pathologists.

The assessment of HIF‐1α and VEGF were based on the previous study studies.^16^, ^17^ Tumour cell immunoreactivity for HIF‐1α was scored according to the nuclear staining. Both percentage of positive stained tumour cells and the staining intensity were taken into account to determine the expression of HIF‐1α. The percentage of positive cells was rated as follows: 1 point, ≦10% positive tumour cells; 2 points, 11%‐50% positive cells; 3 points, 51%‐80% positive cells; and 4 points, ≧81% positive cells. The staining intensity was rated as follows: 1 point, weak intensity; 2 points, moderate intensity; and 3 points, strong intensity. The sum of the two parameters varied between 2 and 7. For statistical reasons, tumours were then scored according to a two‐scale system: low reactivity (non‐overexpression) denoting tumours with scoring ≦5points and high reactivity (overexpression) denoting tumours with scoring 6‐7 points. For VEGF immunoreactivity was detected in the cytoplasm of the cells. The IHC score was calculated by adding the percentage of positively stained cells to the staining intensity. The percentage of positive cells ranged between 0 and 3, ie 0, if less than 10% of tumour cells were stained; 1, if 10%‐25% of tumour cells were stained; 2, if 25%‐50% were positive; and 3, if >50% were positive. The staining intensity was scored as: 0, negative immunoreaction; 1, weak intensity; 2, moderate intensity; and 3, strong intensity. The sum of the two parameters varied between 0 and 6. In our study, we considered the statistical convenience and divided into low reactivity (non‐overexpression), scoring ≦4 points and high reactivity (overexpression), scoring 5‐6 points.

### 2.12 Human umbilical vein endothelial cell tube formation assay

Tube formation experiments were performed with an Angiogenesis Starter Kit (Applied Biosystems) according to the manufacturer's manual. Human umbilical vein endothelial cells (HUVECs) seeded on the gel were cultured for 24 hours with the conditioned medium collected from the transfected HCT116 or HT29 cells. Cells were stained using a PKH26 Red Fluorescent Cell Linker Mini Kit (Sigma‐Aldrich Inc) for microscopic visualization. Photographs were obtained using NIS‐Elements imaging software version 3.22.14 connected to the Eclipse Ti‐U inverted microscope system (Nikon Instruments Inc, Melville, NY). After overnight incubation at 37°C, the plates were photographed and the extent of tube formation was assessed.

### 2.13 Luciferase assay

pLightSwith‐HIF‐1α 3ʹUTR (Untranslated regions) luciferase reporter plasmid was purchased from Active Motif (Carlsbad, CA). For transfection, cells were seed to yield 80% confluence in 96 well plates. The next day, cells were co‐transfected with either pLightswitch‐HIF‐1α 3ʹUTR or pLightswitch 3ʹUTR luciferase reporter plasmid and cypridina control construct. Transfections were performance using Lipofectamine 2000 (Thermo Fisher Scientific) following manufacturers instruction. Luciferase activities were measured 48 hours after transfection using the LightSwitch Dual Assay system (Active Motif) by BioTek FLx800 Multi‐Detection Microplate Reader (BioTek, Winooski, VT).

### 2.14 Inhibition of HIF‐1α and VEGF expression by *miR‐148a* in the hypoxic condition

From the previous study demonstrated by Seo et  al, CoCl_2_ is a hypoxia mimetic agent.^18^ Therefore, we used the CoCl_2_ to create a hypoxic culture condition and revealed the ability of inhibition of HIF‐1α and VEGF expression by *miR‐148a* under the hypoxic culture medium.

### 2.15 In vivo animal study

Four‐week‐old Balb/c nude mice (bodyweight 12.6–15.6 g) were purchased from BioLasco Taiwan, Ltd. (Taipei, Taiwan) and maintained in a specific pathogen‐free environment (certificate no. 26‐99S029). At 6 weeks of age, each nude mouse was injected subcutaneously in the neck area with 1 × 10^7^ HCT116 (either NC or *OmiRNA‐148a*; n = 3 per transfected cell line) and 1 × 10^7^ HT29 (either NC or *OmiRNA‐148a*; n = 3 per transfected cell line). Mouse weight and tumour volume (V) were measured every Monday, Wednesday and Friday. Volume was calculated using the following formula: V = 0.5 × length (mm) × width^2^ (mm^2^).^19^ The animals were killed 3 weeks after the tumour cells had been seeded. Tumour burdens were analysed and counted immediately without prior fixation.

### 2.16 Statistical analysis

A chi‐square test was used to analyse differences between the two groups (early relapse vs non‐early relapse). Data are presented as the mean ± SD of three independent experiments. All statistical analyses were performed with the Statistical Package for the Social Sciences 19.0 (spss Inc, Chicago, IL). A two‐tailed *P* < 0.05 was considered statistically significant.

## 3 RESULTS

### 3.1 *miRNA‐148a* inhibited the activation of pERK and HIF‐1α

**Figure 2:**
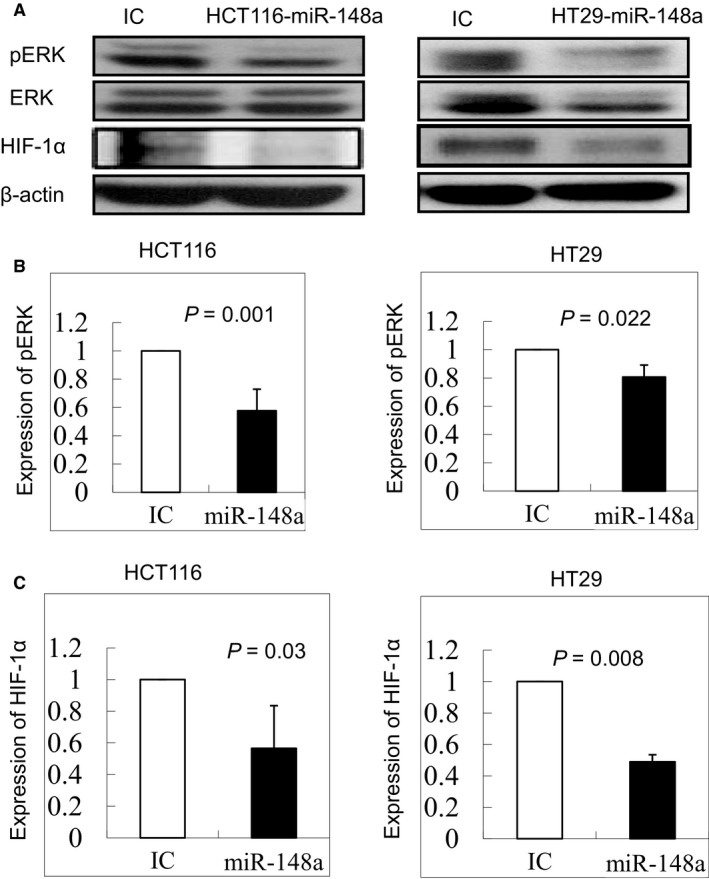
The protein levels of phosphoryl‐ERK (pERK) and hypoxia‐inducible factor‐1α (HIF‐1α) in HCT116 and HT29 cell lines were examined through Western blotting. A, The protein levels of pERK and HIF‐1α were significantly decreased under overexpression of *miR‐148a*. (Full‐length blots/gels are presented in Figure S4). B, The protein expression of pERK was significantly down‐regulated in HCT116 and HT29 cell lines (*P* = 0.001 and 0.022 respectively). C, The protein expression of HIF‐1α was significantly inhibited in HCT116 and HT29 cell lines (*P* = 0.03 and 0.008 respectively)

To investigate the effect of *miRNA‐148a* on the activation of ERK, we demonstrated that the protein level of pERK was prominently suppressed in the HCT116 and HT29 colon cell lines that expressed *miR‐148a *(Figure 2A,B; *P* = 0.001 and 0.022 respectively). In addition, the protein levels of HIF‐1α expression (Figure 2A,C; *P* = 0.03 and 0.008 respectively) were suppressed, indicating that *miR‐148a* might inhibit HIF‐1α expression and its functionality by decreasing downstream pERK activation.

### 3.2 HIF‐1α acts as an indirect target of *miR‐148a*

**Figure 3:**
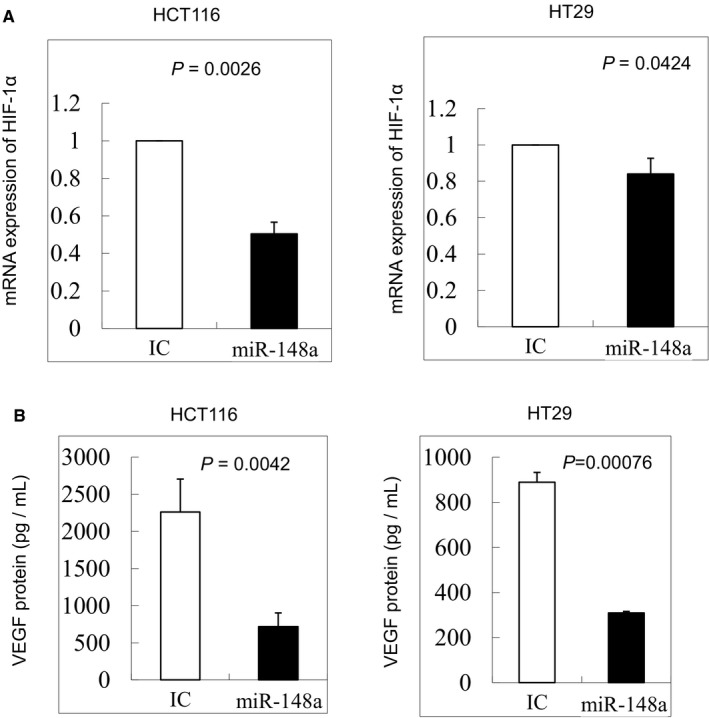
A, *miR‐148a* indirectly targeted hypoxia‐inducible factor‐1α (HIF‐1α) in HCT116 and HT29 cells. *miR‐148a* significantly inhibited the mRNA expression of HIF‐1α in HCT116 and HT29 cells (*P* = 0.0026 and 0.0424 respectively). B, vascular endothelial growth factor (VEGF) secretion was significantly inhibited by *miR‐148a* in HCT116 and HT29 cells, as detected through ELISA (*P = *0.0042 and 0.000 76 respectively)

To determine whether HIF‐1α was a target gene of *miR‐148a*, we compared the mRNA and protein levels of HIF‐1α between cells with *miR‐148a* overexpression and non‐overexpression. The overexpression of *miR‐148a* greatly decreased HIF‐1α expression in the HCT116 and HT29 cell lines, as detected using PCR (Figure 3A; *P* = 0.0026 and 0.0424 respectively) and Western blotting (Figure 2C; *P* = 0.03 and 0.008 respectively), suggesting that HIF‐1α is a downstream target gene of *miR‐148a*. Furthermore, we also demonstrated that HIF‐1α was not directly target gene of *miR‐148a* using the luciferase assay in the both colon cancer cell lines (Figure S2).

### 3.3 Expression of *miR‐148a* curbs VEGF secretion in CRC cell lines under hypoxic and no hypoxic conditions

The secretion levels of VEGF were examined through ELISA in the colon cancer cell lines, HCT116 and HT29. VEGF secretion was significantly down‐regulated in the two CRC cell lines compared with in the control (Figure 3B; *P* = 0.0042 and *P* = 0.000 76), suggesting that VEGF secretion is inhibited by the overexpression of *miR‐148a*.

In the hypoxic culture condition, we also revealed that *miR‐148a* could significantly inhibit the expressions of HIF‐1α and VEGF in HCT116 (*P* = 0.0007 & 0.02 respectively; Figure S3A) and in HT29 (*P* = 0.045 & 0.02 respectively; Figure S3B).

### 3.4 Expression of *miR‐148a* curbs VEGF expression in CRC tissue samples

**Figure 4:**
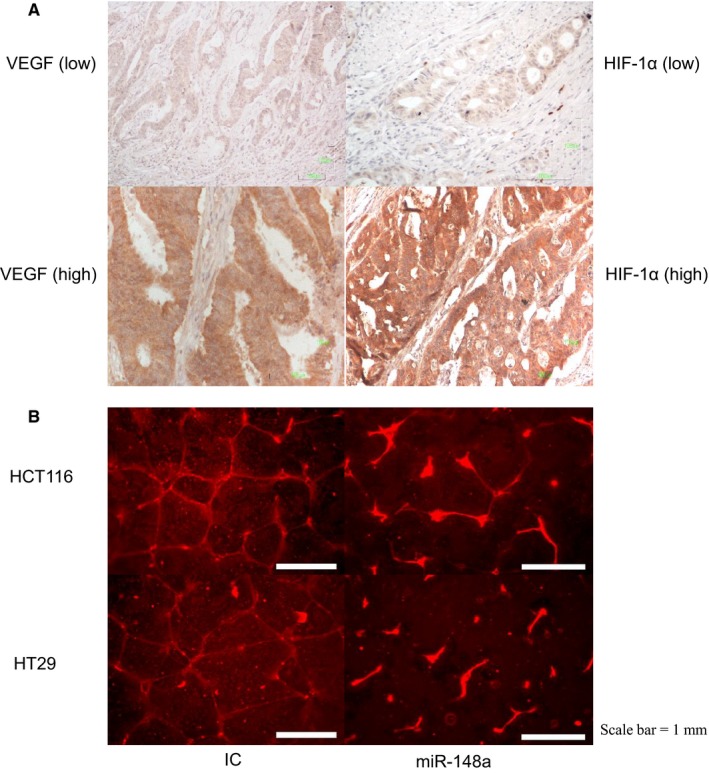
The role of *miR‐148a* in angiogenesis. A, vascular endothelial growth factor (VEGF) and hypoxia‐inducible factor‐1α (HIF‐1α) proteins were stained in the cytoplasm of tumour cells (shown in brown) and assessed through immunohistochemistry staining. *miR‐148a* suppressed the expression of VEGF and HIF‐1α. Left panel shows VEGF (top: low expression; bottom: high expression; 200X). Right panel shows HIF‐1α (top: low expression; bottom: high expression; 200X). B, *miR‐148a* significantly destroyed human umbilical vein endothelial cells tube formation in the HCT116 (top) and HT29 (bottom) cell lines

The demographic data of the patients enrolled in this study are presented in Table 1. A strong inverse correlation was observed between *miR‐148a* expression levels and HIF‐1α and VEGF expression in CRC tissue samples, as evaluated through IHC staining (Table 2; *P* = 0.002 and 0.004 respectively). The overexpression of *miR‐148a* reversed the protein expression of VEGF and HIF‐1α in the tissue samples, as detected through IHC staining (Figure 4A). These results demonstrate that overexpression of *miR‐148a* prominently inhibits VEGF expression in vitro and in vivo.

**Table 1:** Clinicopathologic features of 63 enrolled colorectal cancer patients

Number of patients	Early relapsed with *miR‐148a* non‐overexpression	Non‐early relapsed with *miR‐148a* overexpression	*P*‐value
	28 (%)	35 (%)	
Gender			0.645
Male	16 (57.2)	22 (62.8)	
Female	12 (42.8)	13 (37.2)	
Age (y/o)			0.759
≧65	19 (67.8)	25 (71.4)	
<65	9 (32.2)	10 (28.6)	
Location			0.271
Colon	19 (67.8)	28 (80.0)	
Rectum	9 (32.2)	7 (20.0)	
Stage			0.819
II	16 (57.2)	21 (60.0)	
III	12 (42.8)	14 (40.0)	
Tumour size (cm)			0.260
≧5	16 (57.2)	15 (42.9)	
<5	12 (42.8)	20 (57.1)	
Tumour depth			0.354
T2	0 (0)	2 (5.8)	
T3	26 (92.8)	29 (82.6)	
T4	2 (7.2)	4 (11.6)	
Vascular invasion			0.605
Yes	8 (28.6)	8 (22.9)	
No	20 (71.4)	27 (77.1)	
Perineural invasion			0.085
Yes	8 (28.6)	4 (11.5)	
No	20 (71.4)	31 (88.5)	
Tumour grade			0.469
MD	23 (82.1)	31 (88.5)	
PD	5 (17.9)	4 (11.5)	
Histology			0.466
A	25 (89.2)	33 (94.2)	
M	3 (10.8)	2 (5.8)	

A, adenocarcinoma; M, mucinous carcinom; MD, moderately differentiated; PD, poorly differentiated.

**Table 2:** Relationship between HIF‐1α/VEGF expressions and *miR‐148a* in the 63 CRC patients by using immunohistochemical staining

	*miR‐148a* expression		*P*‐value
	Non‐overexpression (%)	Overexpression (%)	
Number of patients	28	35	
HIF‐1α			0.002
Non‐overexpression	14 (50.0)	30 (85.7)	
Overexpression	14 (50.0)	5 (14.3)	
VEGF			0.004
Non‐overexpression	6 (21.4)	20 (57.1)	
Overexpression	22 (78.6)	15 (42.9)	

CRC, colorectal cancer; HIF‐1α, hypoxia‐inducible factor‐1α; VEGF, vascular endothelial growth factor.

### 3.5 HUVEC tube formation assay

The HUVEC tube formation assay revealed that *miR‐148a* inhibited angiogenesis in both CRC cell lines (Figure 4B). The existence of *miR‐148a* could obliterate the interlocking of vessels. These results demonstrated that overexpression of *miR‐148a* inhibits vein cell tube formation. The results of angiogenesis are actually not quantitative although many statistical charts are included in published literature. The angiogenesis measurement is largely dependent on software setting and measurement changes with setting change. They are better to be considered as visible results as long as difference can be seen between two pictures. However, the statistical charts can be good adjuvant data (*P* = 0.02 in HCT116 cell line and *P* = 0.03 in HT29 cell line; Figures S4 and S5).

### 3.6 In vivo animal study: Effects of *miR‐148a* overexpression in nude mice

To validate the role of *miR‐148a* in tumourigenesis, we determined the effects of *miR‐148a* overexpression on tumour growth in vivo. The *OmiR‐148a* and NC clones with scrambled pCDH‐NC were injected subcutaneously to induce tumour growth in nude mice. The tumours became palpable 7 days after inoculation and were allowed to grow until the end of the third week. Mice that received *OmiR‐148a *cells had significantly smaller cancer lumps than those that received pCDH‐NC cells either in HCT116 or HT29. This in vivo result provides additional support that overexpression of *miR‐148a* results in less tumour cell proliferation in experimental animals (*P* = 0.0007 and 0.0037 in the HCT116 and HT29 cell lines respectively; Figure S6A,B).

## 4 DISCUSSION

A novel finding of the present study is that *miR‐148a* can inhibit the secretion of VEGF through the indirect down‐regulation of HIF‐1α and its relevant pathways. Even under hypoxic condition, we also confirmed that *miR‐148a* efficiently inhibited the expression of HIF‐1α and VEGF. Regardless of in vitro or in vivo conditions, we re‐verified that *miR‐148a* has the ability to inhibit angiogenesis in CRC.

Angiogenesis is a complex process through which new blood vessels are formed from an endothelial precursor. It is a critical step in cancer progression and is considered one of the hallmarks of cancer.^1^ This process is mediated through a group of ligands and receptors that are tightly regulated.^20^, ^21^ Hypoxia‐inducible factor‐1α and VEGF are important regulators of angiogenesis.^22^ Hypoxia‐inducible factor‐1α activates expression of the VEGF gene by binding to the hypoxia response element in the VEGF promoter region.^23^ Hypoxia‐inducible factor‐1α and VEGF are major regulators of angiogenesis^24^ and are important in tumour progression.^25^ As small non‐coding RNAs, *miRNAs* play a crucial role in CRC tumourigenesis and progression^26^ and perform their functions by modulating the expression of their target genes. We previously demonstrated that *miR‐148a* inhibits the early relapse of CRC and resulted in reduced tumour growth in animal model of CRC^11^ and that VEGF can promote early relapse.^27^ In 2013, Xu et  al demonstrated that down‐regulation of *miR‐148a *activated the ERK signalling pathway to increase HIF‐1α and VEGF expression as well as tumour growth and angiogenesis in breast cancer cell lines.^9^ Similarly, we demonstrated that overexpression of *miR‐148a* can down‐regulate pERK to inhibit HIF‐1α and VEGF secretion in colon cancer cell lines. Moreover, by using tumour tissue samples from CRC patients, we certified that the *miRNA‐148a* level is inversely related to the expression of HIF‐1α and VEGF. The mechanism of *miR‐148a*‐mediated suppression of pERK probably explained that certain growth factors activate RAS which in turn stimulates the RAS/RAF/MEK/ERK kinase cascade. Activated ERK phosphorylates 4E‐BP1, S6K and MAP kinase interacting kinase (MNK). MNK can also phosphorylate eIF‐4E directly. The net result of these signalling events is the increased rate of mRNA translation into HIF‐1α protein. Interestingly, ERK is not only involved in regulation of HIF‐1α synthesis but also its transcriptional activation. ERK phosphorylates the co‐activator CBP/p300, so it increases HIF‐1α/p300 complex formation and thus stimulates its transcriptional activation function.^28^

In recent years, many studies have revealed that the aberrant expression of miRNA is closely related to oncogenesis and this is now an intense field of study. *miR‐148a *is aberrantly expressed in various cancers and has been identified as an oncogenic or tumour suppressor with crucial roles in the molecular mechanisms of oncogenesis.^8^ In the some studies, it was also proven that *miR148a*‐mediated suppression of tumour growth and tumour vascular formation existed in vivo experiments.^9^, ^11^ Overexpression of *miR‐148a* was reported to inhibit ERBB3 expression, block downstream pathway activation (including the activation of AKT, ERK1/2 and p70S6K1) and decrease HIF‐1α expression in breast cancer cell lines.^29^ Some VEGF‐targeted *miRNAs*, including *miR‐93*,^2^ *miR‐203*,^30^ *miR‐26a*,^31^ *miR‐497*,^32^ *miR‐199a‐5p* 33 and *miR‐140‐5p* 26 have an antitumour effect in various human cancers. According to several algorithms, we initially suggested that that *miR‐148a* targets HIF‐1α to down‐regulate VEGF. In the present study, we demonstrated that overexpression of *miR‐148a* significantly reduced VEGF protein secretion in vitro and in vivo through the inhibition of HIF‐1α. VEGF expression had a remarkable inverse correlation with *miR‐148a* expression in CRC tissue and cell lines. This regulation is independent of ERK modulation by *RAS/RAF* pathway. Therefore, the effects of *RAS/RAF* mutation on ERK regulation in HCT116 and HT29 cells may be bypassed. Finally, we suggested that *miR‐148a* down‐regulates VEGF through the pERK/HIF‐1α pathway in CRC and might be closely associated with early relapse of CRC. However, other upstream pathways for ERK regulation may be also considered and further studies should be carried out.

The cytokine VEGF is an angiogenic factor implicated in processes such as organ development, wound healing, tissue regeneration, endothelial cell growth and vessel permeability.^34^ In some solid tumours, overexpression of VEGF is associated with increased angiogenesis, growth and/or metastasis.^35^, ^36^ Researchers have also demonstrated that VEGF is not only a promising therapeutic target but also seems to be a poor prognostic factor for several cancers.^37^, ^38^, ^39^ In CRC, VEGF‐signalling‐induced neovascularity is a key mediator of tumour angiogenesis, invasion and dissemination.^40^ VEGF level is increased in CRC and associated with a malignancy's increased ability to spread and a poorer prognosis.^38^, ^41^ Previously, we demonstrated that VEGF played an important role in the post‐operative early relapse of CRC patients, following radical resection.^27^ In the current study, we further demonstrated a negative correlation between *miR‐148a* and VEGF expression or secretion in the corresponding colorectal tissues and CRC cancer cell lines.

In conclusion, our study revealed that *miR‐148a* down‐regulated VEGF through the pERK/HIF‐1α pathway. Through the inhibition of VEGF, overexpression of *miR‐148a* might reduce post‐operative early relapse in CRC patients. By using the informatics analysis and luciferase assay, we found that HIF‐1α is not a potential directly target for *miR‐148a*. Therefore, we believe that *miR‐148a* does not directly bind to the 3ʹ‐UTR region of HIF‐1α, but by inhibiting the expression of other genes. However, we would certainly take into consideration of investigating the direct target genes and the mechanism of reduced ERK phosphorylation in our future studies.

## RESEARCH ETHICS

This study has been conducted in accordance with ethical standards and according to the Declaration of Helsinki and the national and international guidelines and has been approved by the authors' institutional review board. All processes involving the patients were approved by the Institutional Review Boards of Kaohsiung Medical University Hospital (KMUH).

## CONFLICT OF INTEREST

The authors declare no competing financial interests.

## AUTHOR CONTRIBUTION

HL Tsai carried out the study design, molecular genetic studies, participated in the sequence alignment and drafted the manuscript. ZF Miao was responsible for molecular genetic studies and study design. YT Chen was carried out the IHC study. CW Huang and YS Yeh were responsible for data collection and analysed the data. JY Wang conceived of the study and participated in its design and coordination. All authors read and approved the final manuscript.

## References

[1] Mousa L , Salem ME , Mikhail S . Biomarkers of angiogenesis in colorectal cancer. Biomark Cancer. 2015;7:13‐19. 10.4137/BIC.S25250 PMC4624093 26543385

[2] Yang I‐P , Tsai H‐L , Hou M‐F , et al. MicroRNA‐93 inhibits tumor growth and early relapse of human colorectal cancer by affecting genes involved in the cell cycle. Carcinogenesis. 2012;33:1522‐1530. 22581829 10.1093/carcin/bgs166

[3] Tsai HL , Chu KS , Huang YH , et al. Predictive factors of early relapse in UICC stage I‐III colorectal cancer patients after curative resection. J Surg Oncol. 2009;100:731‐743. 10.1002/jso.21404 19757443

[4] Hedge SR , Sun W , Lynch JP . Systemic and targeted therapy for advanced colon cancer. Expert Rev Gastroenterol Hepatol. 2008;2:135‐149. 19072376 10.1586/17474124.2.1.135

[5] Sibley CR , Seow Y , Wood MJ . Novel RNA‐based strategies for therapeutic gene silencing. Mol Ther. 2010;18:466‐476. 20087319 10.1038/mt.2009.306 PMC2839433

[6] Guo P , Coban O , Snead NM , et al. Engineering RNA for targeted siRNA delivery and medical application. Adv Drug Deliv Rev. 2010;62:650‐666. 20230868 10.1016/j.addr.2010.03.008 PMC2906696

[7] Kim SJ , Oh JS , Shin JY , et al. Development of microRNA‐145 for therapeutic allocation in breast cancer. J Control Release. 2011;155:427‐434. 21723890 10.1016/j.jconrel.2011.06.026

[8] Li Y , Deng X , Zeng X , Peng X . The role of miR‐148a in cancer. J Cancer. 2016;7:1232‐1241. 10.7150/jca.14616 PMC4934031 27390598

[9] Xu Q , Jiang Y , Yin Yu , et al. A regulatory circuit of miR‐148a/152 and DNMT1 in modulating cell transformation and tumor angiogenesis through IGF‐IR and IRS1. J Mol Cell Biol. 2013;5:3‐13. 22935141 10.1093/jmcb/mjs049 PMC3570052

[10] Mizukami Y , Fujiki K , Duerr E‐M , et al. Hypoxic regulation of vascular endothelial growth factor through the induction of phosphatidylinositol 3‐kinase/Rho/ROCK and c‐Myc. J Biol Chem. 2006;281:13957‐13963. 16543245 10.1074/jbc.M511763200

[11] Tsai HL , Yang IP , Huang CW , et al. Clinical significance of microRNA‐148a in patients with early relapse of stage II and III colorectal cancer after curative resection. Transl Res. 2013;162:258‐268. 23933284 10.1016/j.trsl.2013.07.009

[12] Eilken HM , Adams RH . Turning on the angiogenic microswitch. Nat Med. 2010;16:853‐854. 20689545 10.1038/nm0810-853

[13] Semenza GL . Regulation of hypoxia‐induced angiogenesis: A chaperone escorts VEGF to the dance. J Clin Invest. 2001;108:39‐40. 11435455 10.1172/JCI13374 PMC209344

[14] Dweep H , Gretz N . miRWalk 2.0: a comprehensive atlas of microRNA‐target interactions. Nat Methods. 2015;12:697 10.1038/nmeth.3485 26226356

[15] Hwang CC , Chai HT , Chen HW , et al. S100B protein expression as an independent predictor of early relapse in UICC stage II and III colon cancer patients after curative resection. Ann Surg Oncol. 2011;18:139‐145. 20628824 10.1245/s10434-010-1209-7

[16] Theodoropoulos VE , Lazaris ACh , Sofras F , et al. Hypoxia‐inducible factor‐1 expression correlates with angiogenesis and unfavorable prognosis in bladder cancer. Eur Urol. 2004;46:200‐208. 15245814 10.1016/j.eururo.2004.04.008

[17] Nesreen HH , Neveen ST . Expression of cyclooxygenase 2 and vascular endothelial growth factor in gastric carcinoma: relationship with clinicopathological parameters. J Egypt Natl Canc Inst. 2016;28:149‐156. 27342370 10.1016/j.jnci.2016.05.005

[18] Seo S , Seo K , Ki SH , Shin SM . Isorhamnetin inhibits reactive oxygen species‐dependent hypoxia inducible factor (HIF)‐1α accumulation. Biol Pharm Bull. 2016;39:1830‐1838. 27803454 10.1248/bpb.b16-00414

[19] Chen H , Cong Q , Du Z , et al. Sulfated fucoidan FP08S2 inhibits lung cancer cell growth in vivo by disrupting angiogenesis via targeting VEGFR2/VEGF and blocking VEGFR2/Erk/VEGF signaling. Cancer Letter. 2016;382:44‐52. 10.1016/j.canlet.2016.08.020 27569654

[20] Jain RK . Normalization of tumor vasculature: an emerging concept in antiangiogenic therapy. Science. 2005;307:58‐62. 15637262 10.1126/science.1104819

[21] Hanahan D , Weinberg RA . The hallmarks of cancer. Cell. 2000;100:57‐62. 10647931 10.1016/s0092-8674(00)81683-9

[22] Forsythe J , Jiang BH , Iyer NV , et al. Activation of vascular endothelial growth factor gene transcription by hypoxia‐inducible factor I. Mol Cell Biol. 1996;16:4604‐4613. 8756616 10.1128/mcb.16.9.4604 PMC231459

[23] Semenza GL . Hypoxia, clonal selection, and the role of HIF‐1α in tumor progression. Crit Rev Mol Bio. 2000;35:71‐103. 10.1080/10409230091169186 10821478

[24] Plate KH , Breier G , Weich A , Risau W . Vascular endothelial growth factor in a potential tumor angiogenesis factor in human gliomas in vivo. Nature. 1992;359:845‐848. 1279432 10.1038/359845a0

[25] Ferrara N , Davis‐Smyth T . The biology of vascular endothelial growth factor. Endo Rev. 1997;18:4‐25. 10.1210/edrv.18.1.0287 9034784

[26] Zhang W , Zou C , Pan L , et al. MicroRNA‐140‐5p inhibits the progression of colorectal cancer by targeting VEGFA. Cell Physiol Biochem. 2015;37:1123‐1133. 26402430 10.1159/000430237

[27] Tsai H‐L , Yang I‐P , Lin C‐H , et al. Predictive value of vascular endothelial growth factor overexpression in early relapse of colorectal cancer patients after curative resection. Int J Colorectal Dis. 2013;28:415‐424. 22961433 10.1007/s00384-012-1570-z

[28] Georgina NM , Li W . HIF‐1α pathway: role, regulation and intervention for cancer therapy. Acta Pharmaceutic Sinica B. 2015;5:378‐389. 10.1016/j.apsb.2015.05.007 PMC4629436 26579469

[29] Yu J , Li Qi , Xu Q , Liu L , Jiang B . MiR‐148a inhibits angiogenesis by targeting ERBB3. J Biomed Res. 2011;25:170‐177. 23554686 10.1016/S1674-8301(11)60022-5 PMC3597061

[30] Zhu X , Er K , Mao C , et al. MiR‐203 suppresses tumor growth and angiogenesis by targeting VEGFA in cervical cancer. Cell Physiol Biochem. 2013;32:64‐73. 23867971 10.1159/000350125

[31] Chai ZT , Kong J , Zhu XD , et al. MicroRNA‐26a inhibits angiogenesis by down‐regulating VEGFA through the PIK3C2alpha/AKT/HIF‐1α pathway in hepatocellular carcinoma. PLoS ONE. 2013;8:e77957 10.1371/journal.pone.0077957 24194905 PMC3806796

[32] Wang W , Ren F , Wu Q , Jiang D , Li H , Shi H . MicroRNA‐497 suppresses angiogenesis by targeting vascular endothelial growth factor A through the PI3K/AKT and MAPK/ERK pathways in ovarian cancer. Oncol Rep. 2014;32:2127‐2133. 25176450 10.3892/or.2014.3439

[33] Hsu CY , Hsieh TH , Tsai CF , et al. miRNA‐199‐5p regulates VEGFA in endometrial mesenchymal stem cells and contributes to the pathogenesis of endometriosis. J Pathol. 2014;232:330‐343. 24155090 10.1002/path.4295

[34] Brown LF , Detmar M , Claffey K , et al. Vascular permeability factor/vascular endothelial growth factor: a multifunctional angiogenic cytokine. EXS. 1997;97:233‐269. 10.1007/978-3-0348-9006-9_10 9002222

[35] Slattery ML , Lundgreen A , Wolff RK . VEGFA, FLT1, KDR and colorectal cancer: assessment of disease risk, tumor molecular phenotype, and survival. Mol Carcinog. 2014; 53(Supp 1):E140‐E150. 10.1002/mc.22058 23794399

[36] Goos JA , de Cuba EM , Coupe VM , et al. Glucose transporter (SLC2A1) and vascular endothelial growth factor A (VEGFA) predict survival after resection of colorectal cancer liver metastasis. Ann Surg. 2016;263:138‐1345. 25563886 10.1097/SLA.0000000000001109

[37] Pignot G , Bieche I , Vacher S , et al. Large‐scale real‐time reverse transcription‐PCR approach of angiogenic pathways in human transitional cell carcinoma of the bladder: identification of VEGFA as a major independent prognostic marker. Eur Urol. 2009;56:678‐688. 18513850 10.1016/j.eururo.2008.05.027

[38] Des Guetz G , Uzzan B , Nicolas P , et al. Microvessel density and VEGF expression are prognostic factors in colorectal cancer. Meta‐analysis of the literature. Br J Cancer. 2006;94:1823‐1832. 16773076 10.1038/sj.bjc.6603176 PMC2361355

[39] Chiang DY , Villanueva A , Hoshida Y , et al. Focal gains of VEGF and molecular classification of hepatocellular carcinoma. Cancer Res. 2008;68:6779‐6788. 18701503 10.1158/0008-5472.CAN-08-0742 PMC2587454

[40] Grothey A , Galanis E . Targeting angiogenesis progress with anti‐VEGF treatment with large molecule. Nat Rev Clin Oncol. 2009;6:507‐518. 19636328 10.1038/nrclinonc.2009.110

[41] Ferroni P , Spila A , Martini F , et al. Prognostic value of vascular endothelial growth factor tumor tissue content of colorectal cancer. Oncology. 2005;69:145‐153. 16127285 10.1159/000087838

