# Supplementary material for: miR‐148a inhibits early relapsed colorectal cancers and the secretion of VEGF by indirectly targeting HIF‐1α under non‐hypoxia/hypoxia conditions
Source: J Cell Mol Med. 2019 Mar 4;23(5):3572–82. doi: 10.1111/jcmm.14257 (PMC6484316; doi:10.1111/jcmm.14257)

Supplementary Figure 1A

miR-148a level after pCDH-*miR-148a* vector transfected into HCT116 and HT29

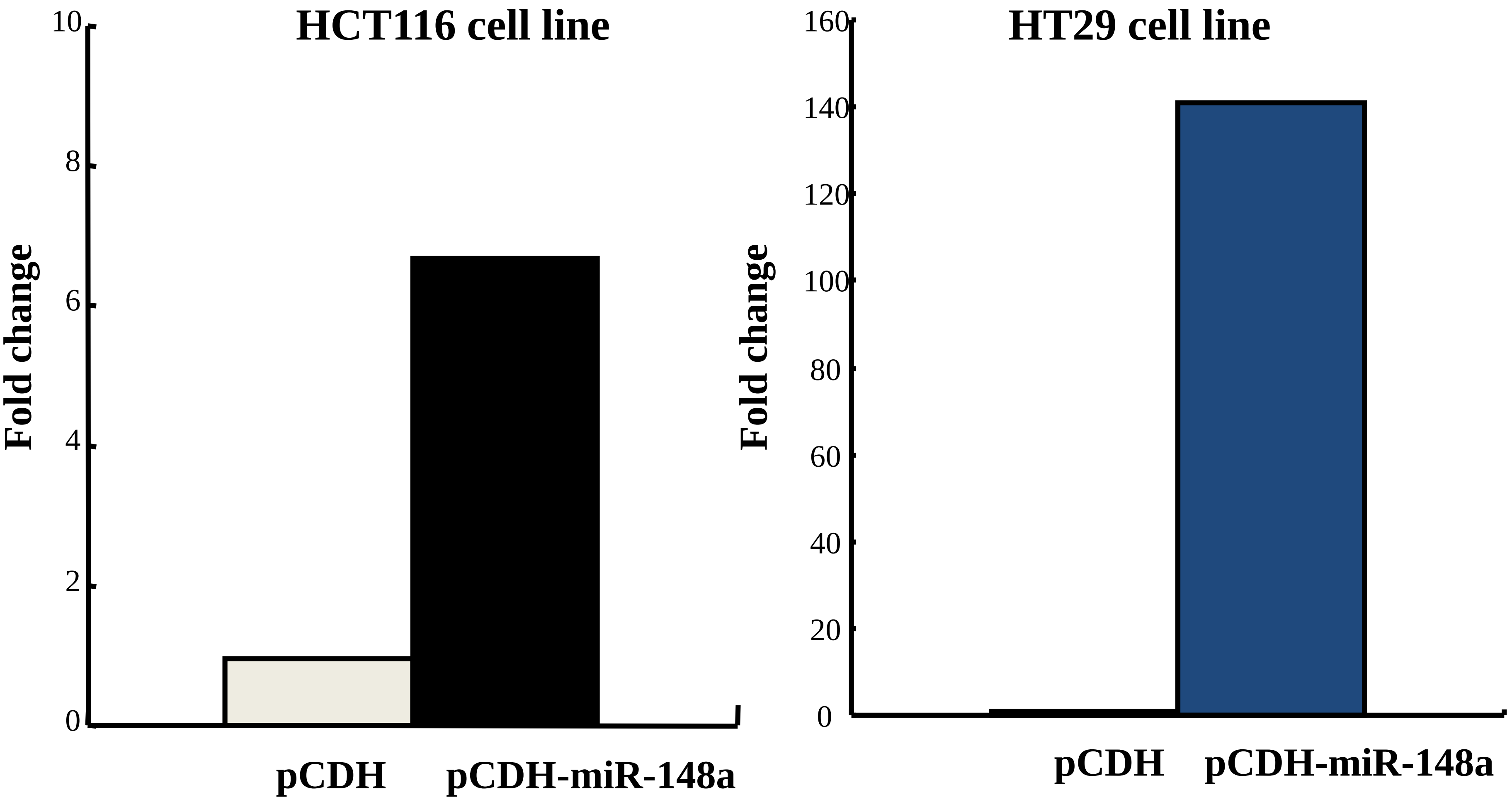

Supplementary Figure 1B

miR-148a level after pCDH-*miR-148a* vector transfected into SW480, SW620, and Caco-2

SW480 cell line

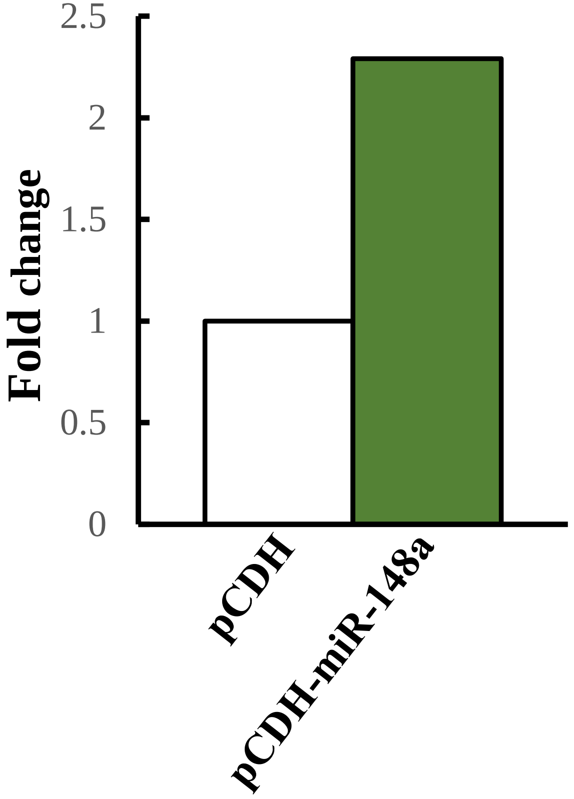

SW620 cell line

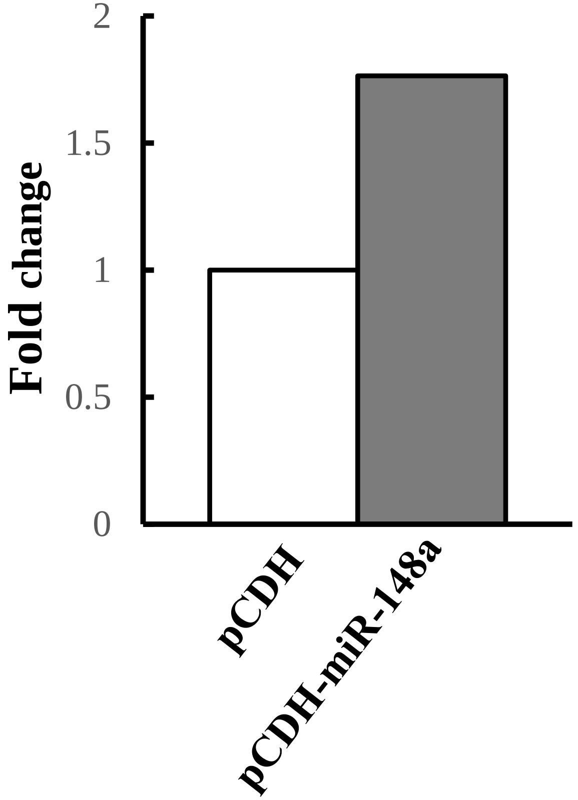

Caco-2 cell line

pCDH-*miR-148a* vector transfected into Caco-2 cell line was not successfully performed. Therefore, constant *miR-148a* level in transfected Caco-2 was not determined.

Supplementary Figure 2

***miR-148a* indirectly inhibits the HIF-1α by luciferase assay in HCT116 and HT29 cell lines**

Reporter assay (Luciferase assay):

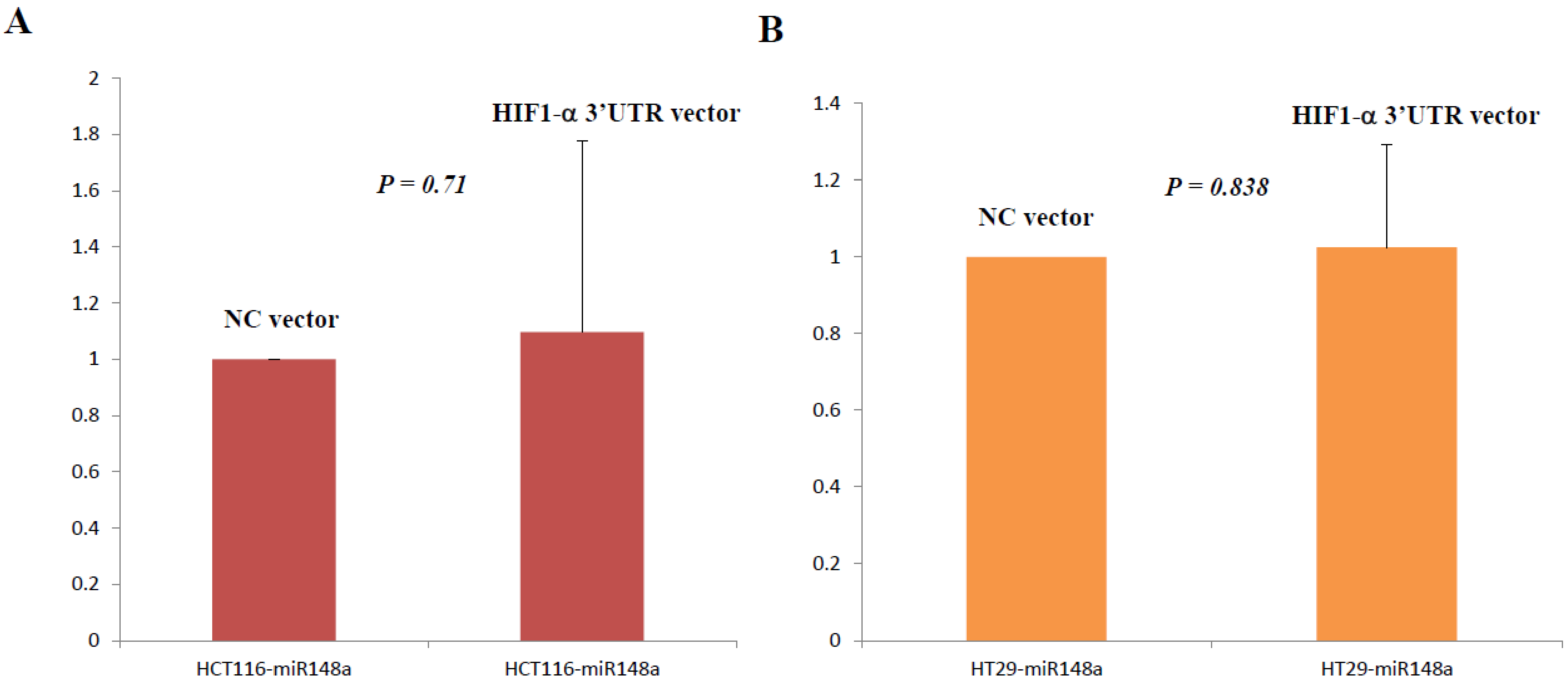

Supplementary Figure 3

A HCT116 (Full-length blots/gels are presented in Supplementary Figure 4A)

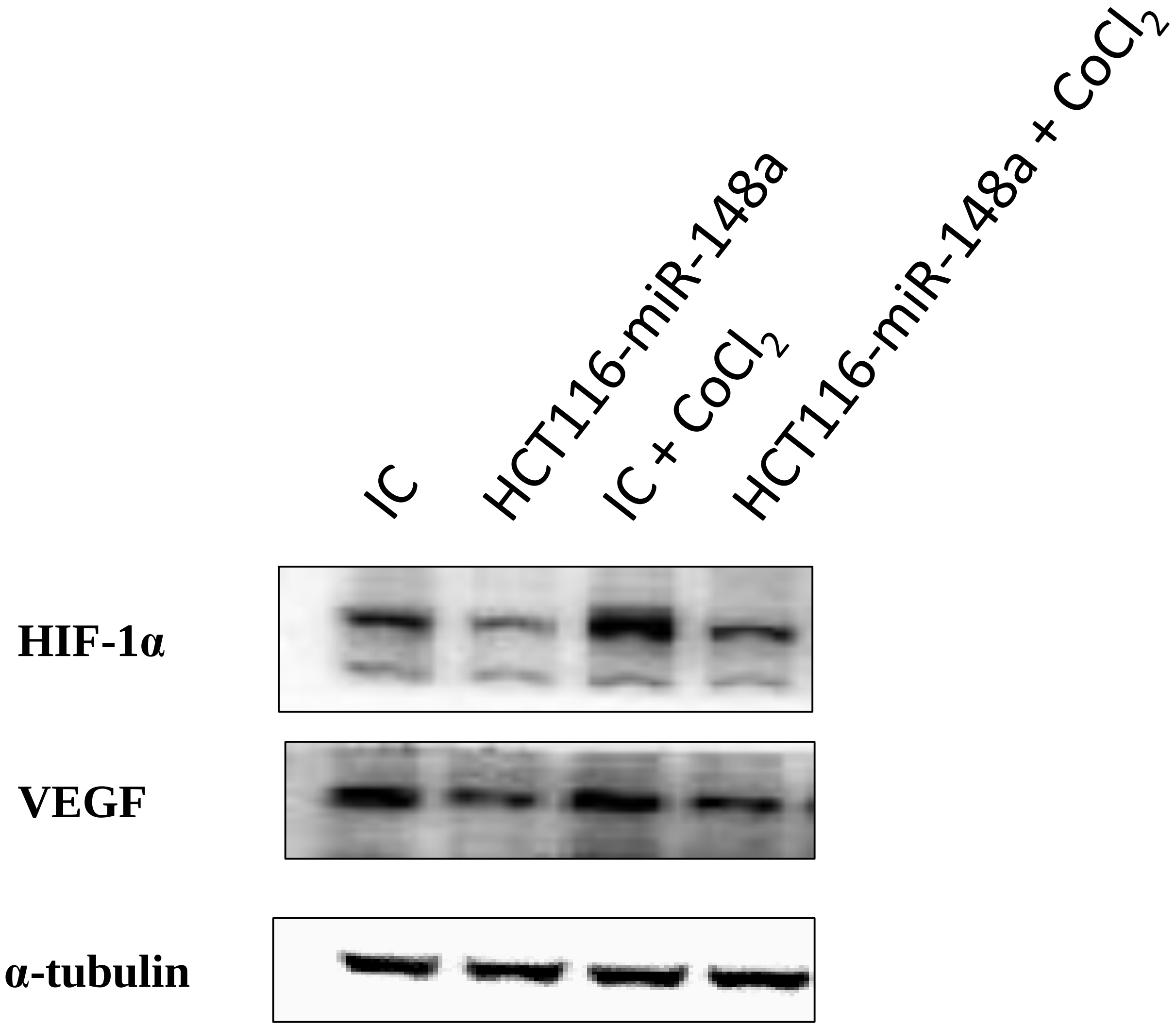

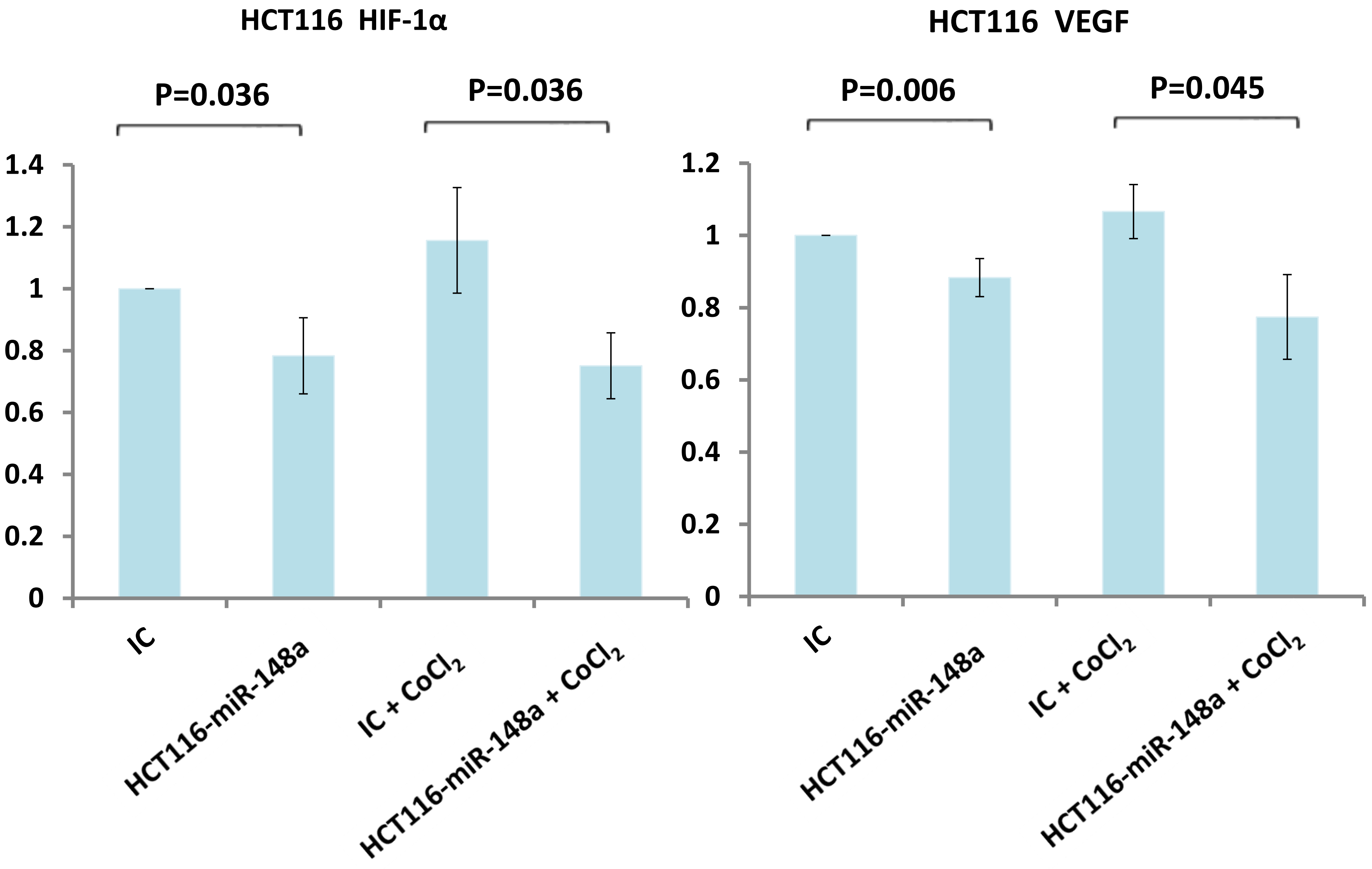
HCT116 HIF-1α
P=0.036
P=0.036
1.4
1.2
1
0.8
0.6
0.4
0.2
0
IC
HCT116-miR-148a
IC + CoCl2
HCT116-miR-148a + CoCl2
HCT116 VEGF
P=0.006
P=0.045
1.2
1
0.8
0.6
0.4
0.2
0
IC
HCT116-miR-148a
IC + CoCl2
HCT116-miR-148a + CoCl2

B HT29 (Full-length blots/gels are presented in Supplementary Figure 4B)

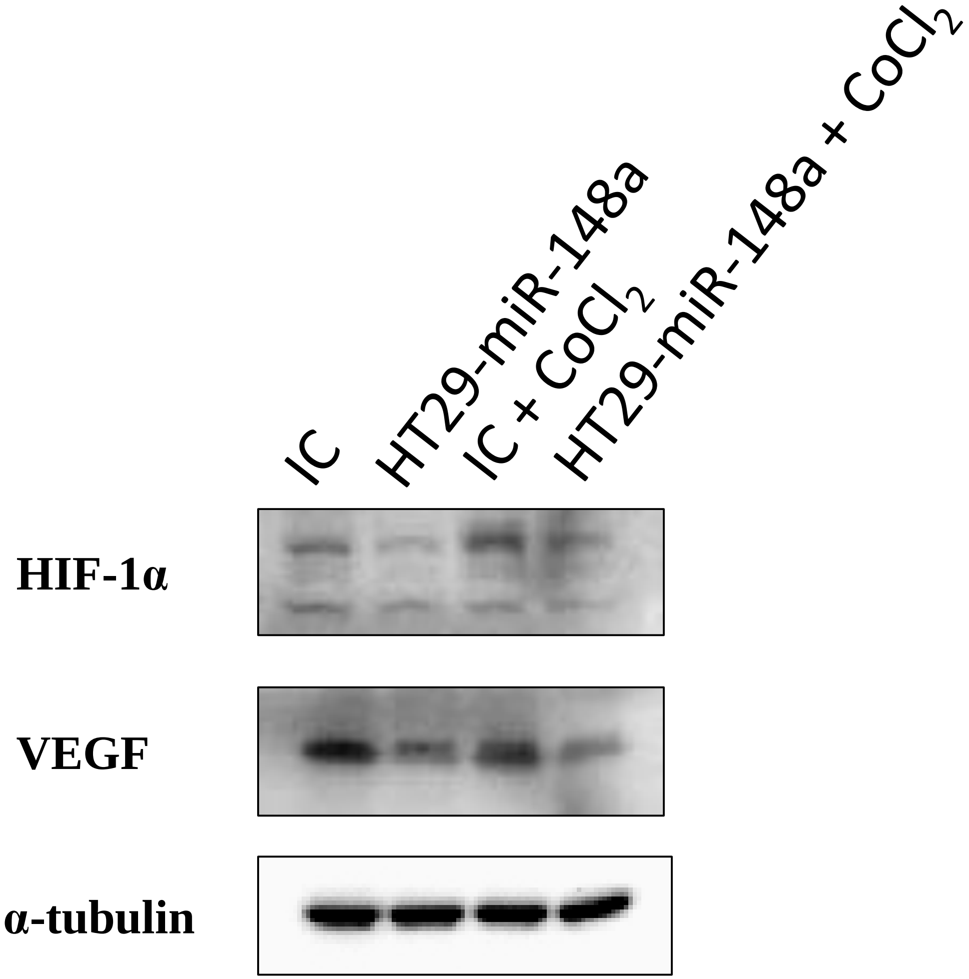

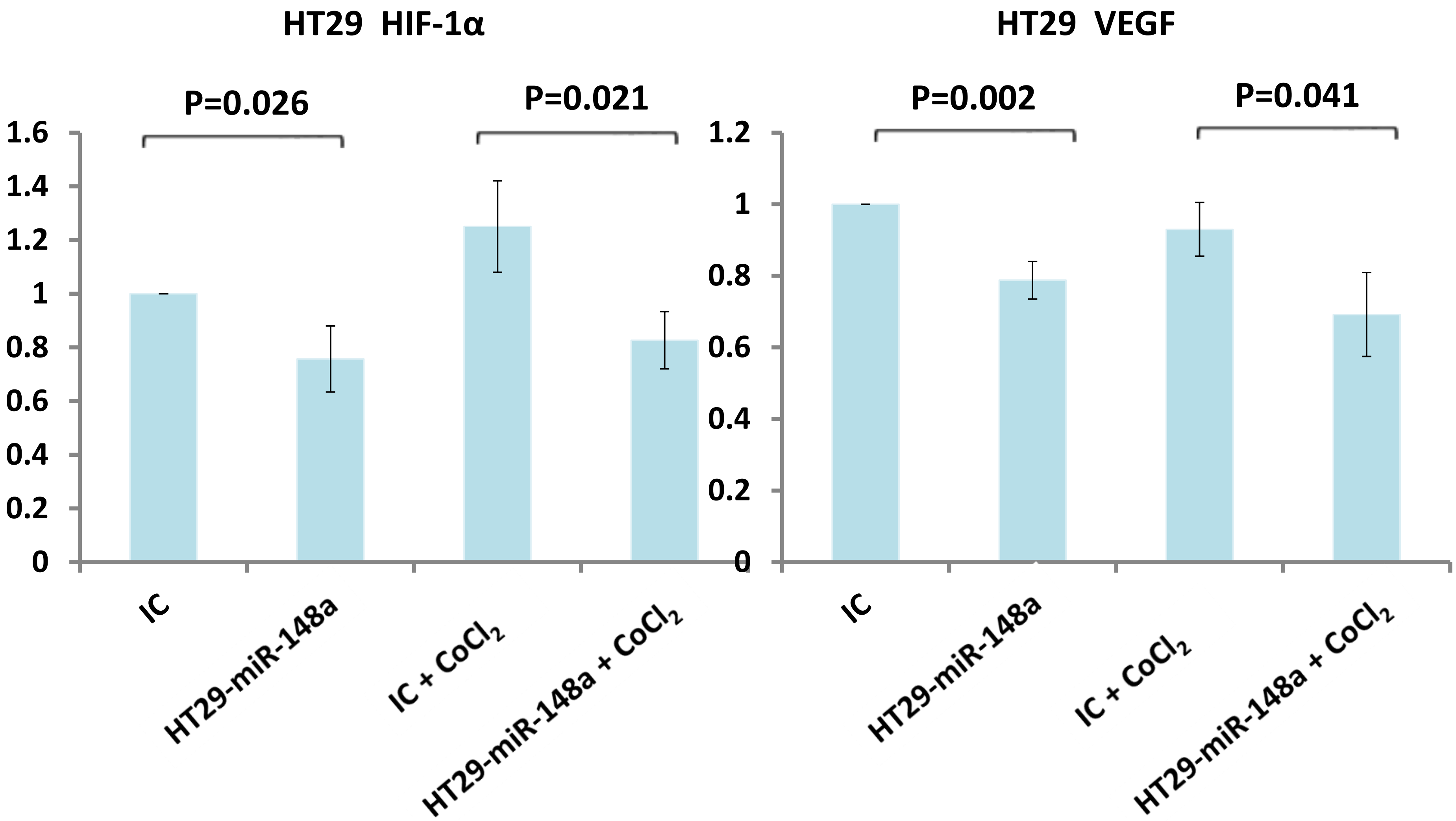
HT29 HIF-1α
P=0.026
P=0.021
1.6
1.4
1.2
1
0.8
0.6
0.4
0.2
0
IC
HT29-miR-148a
IC + CoCl2
HT29-miR-148a + CoCl2
HT29 VEGF
P=0.002
P=0.041
1.2
1
0.8
0.6
0.4
0.2
0
IC
HT29-miR-148a
IC + CoCl2
HT29-miR-148a + CoCl2

Supplementary Figure 4

HUVEC tube formation assay:

A **B**

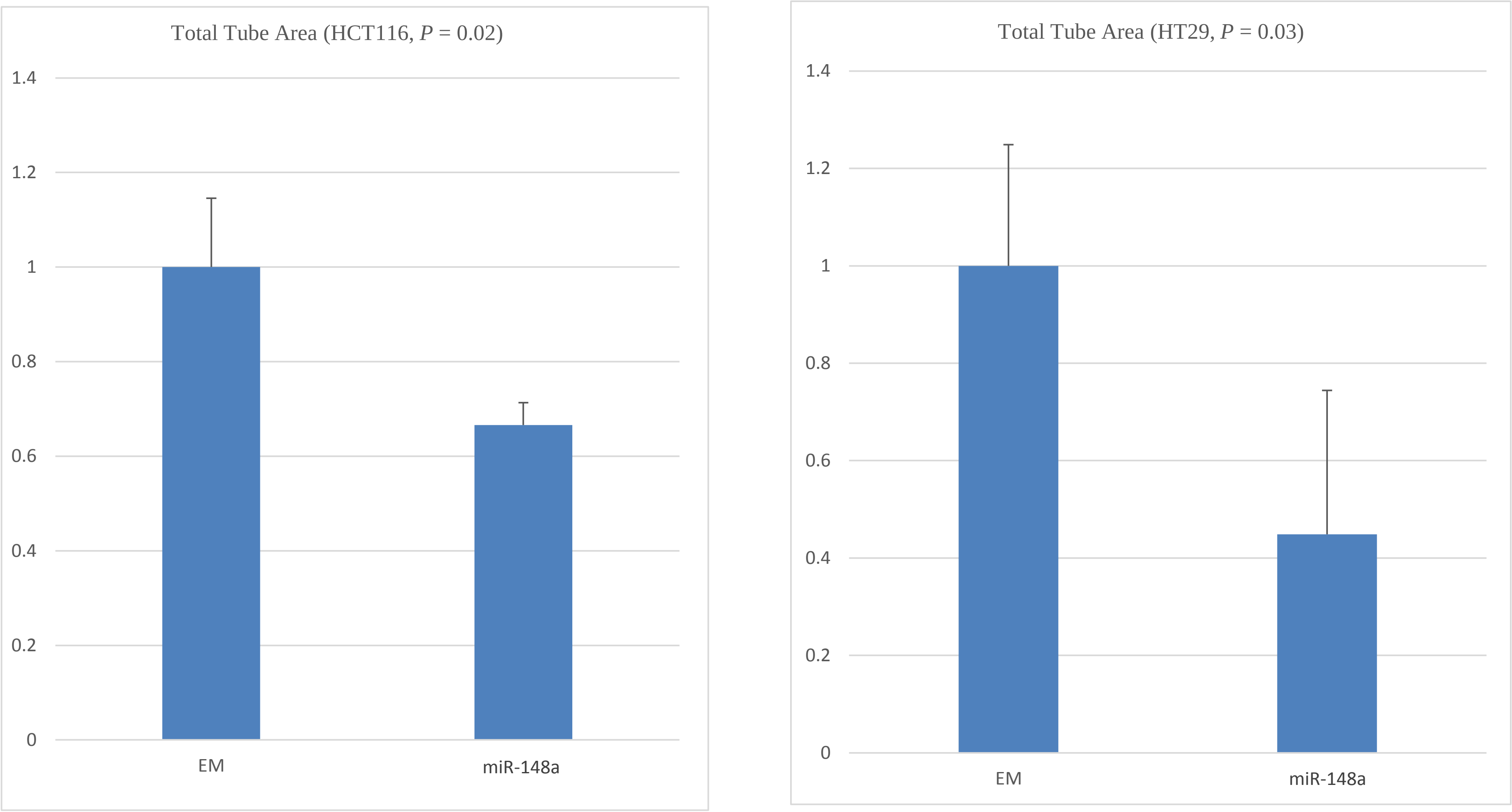

Supplementary Figure 5

A HCT116 (Full-length blots/gels)

HIF-1α

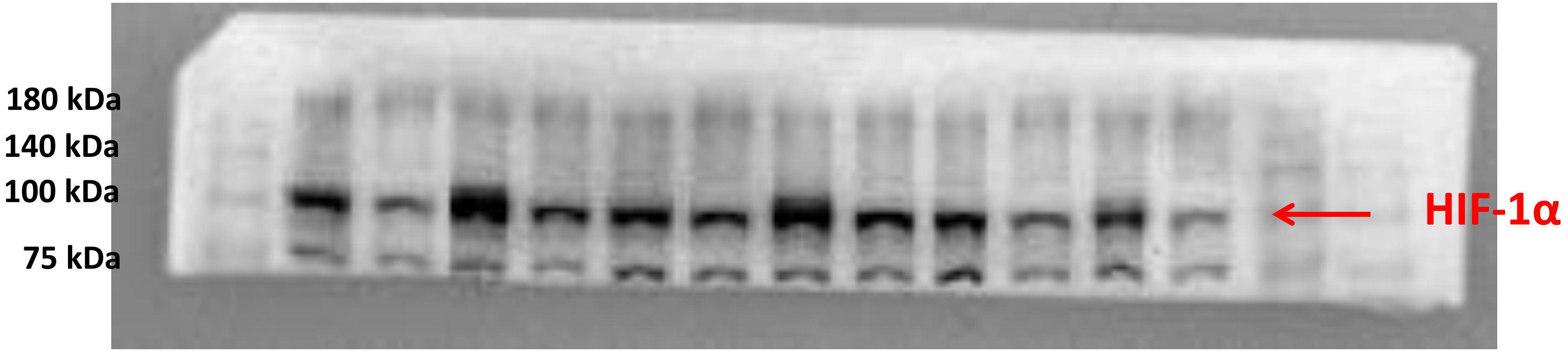

VEGF

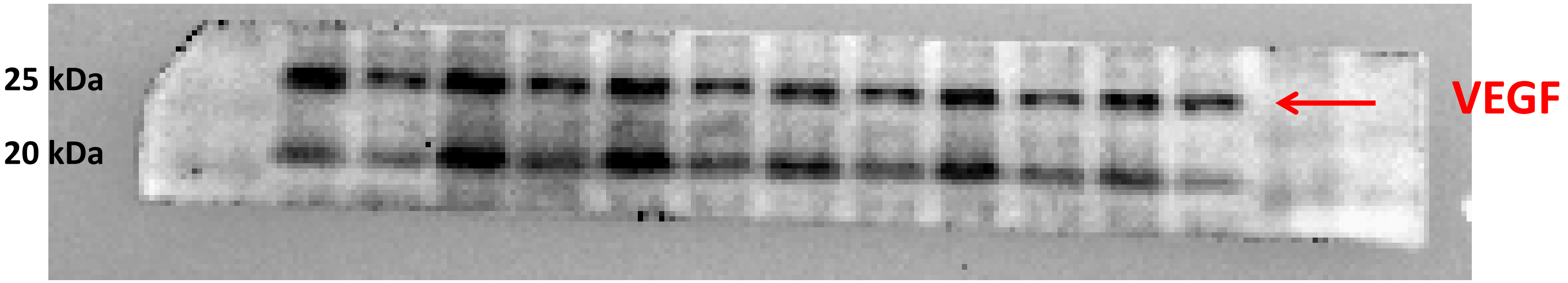

α-tubulin

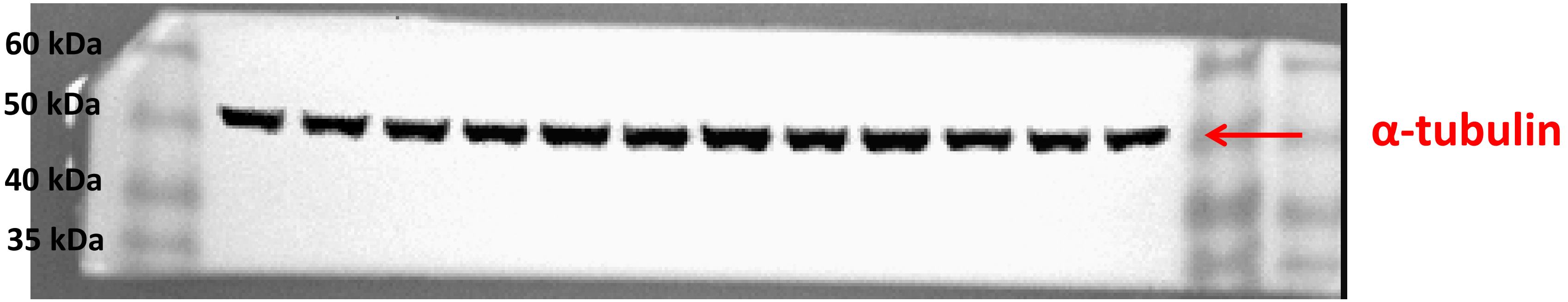

HCT116 (N=3)

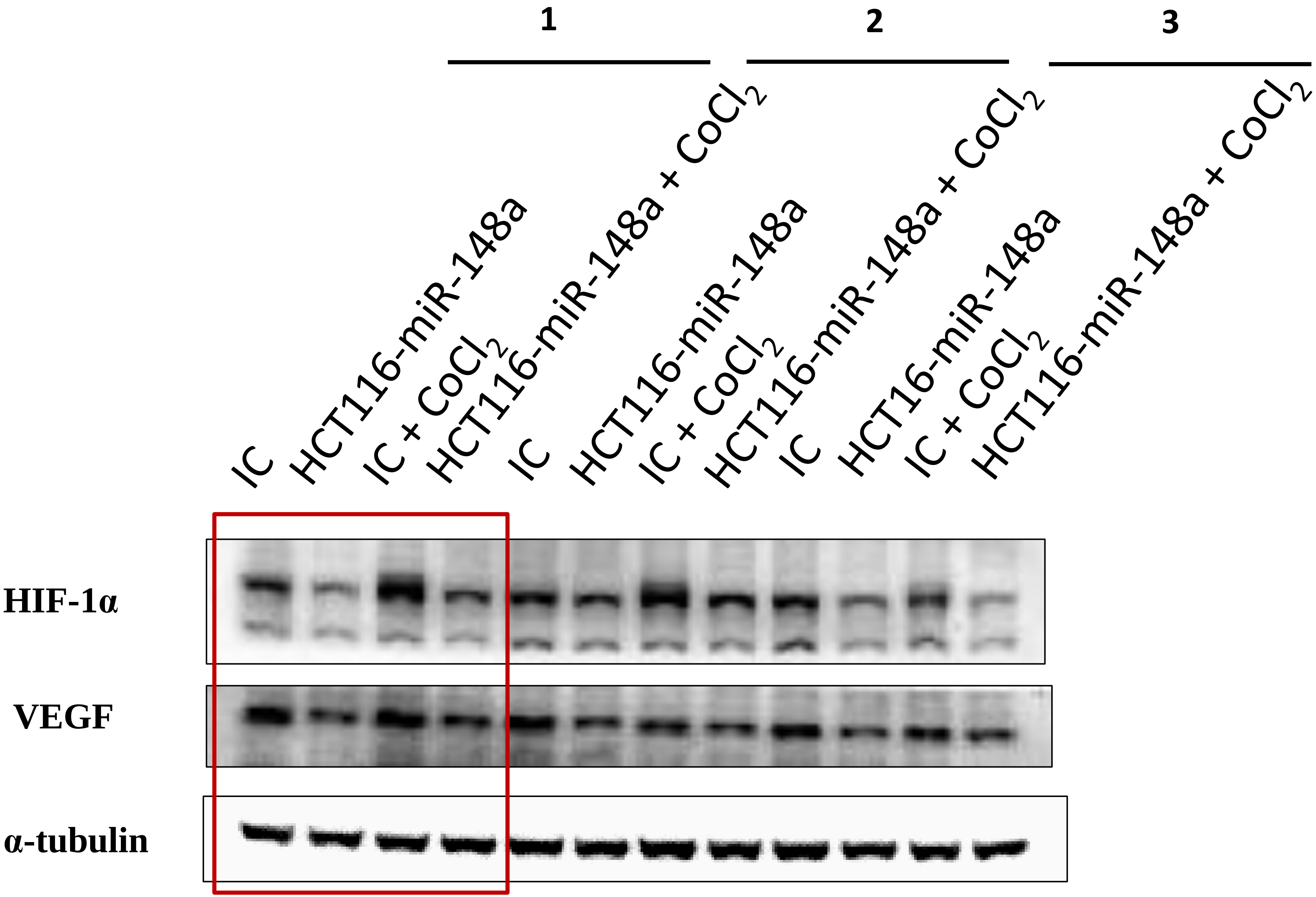
1
2
3
IC
HCT116-miR-148a
IC + $CoCl_2$
HCT116-miR-148a + $CoCl_2$
IC
HCT116-miR-148a
IC + $CoCl_2$
HCT116-miR-148a + $CoCl_2$
IC
HCT16-miR-148a
IC + $CoCl_2$
HCT116-miR-148a + $CoCl_2$
HIF-1α
VEGF
α-tubulin

B HT29 (Full-length blots/gels)

HIF-1α

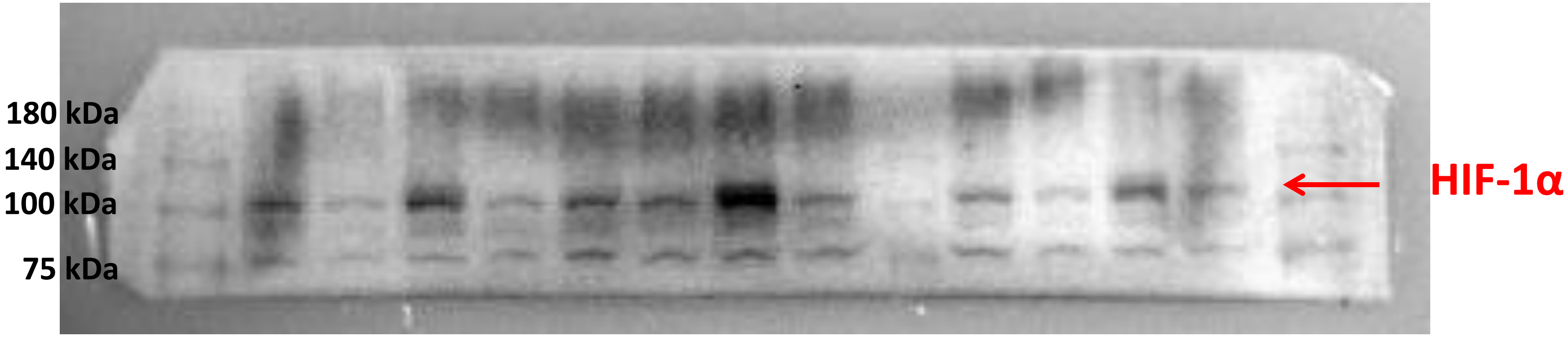

VEGF

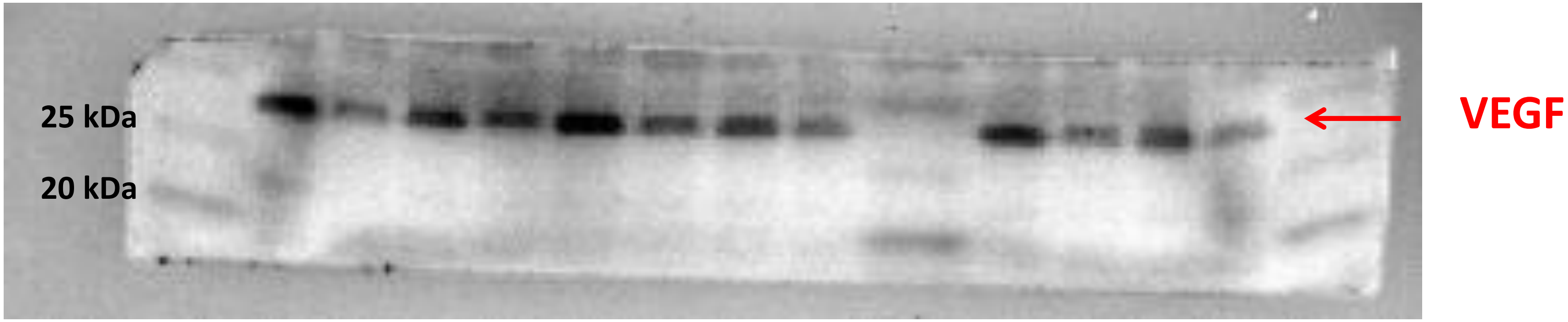

α-tubulin

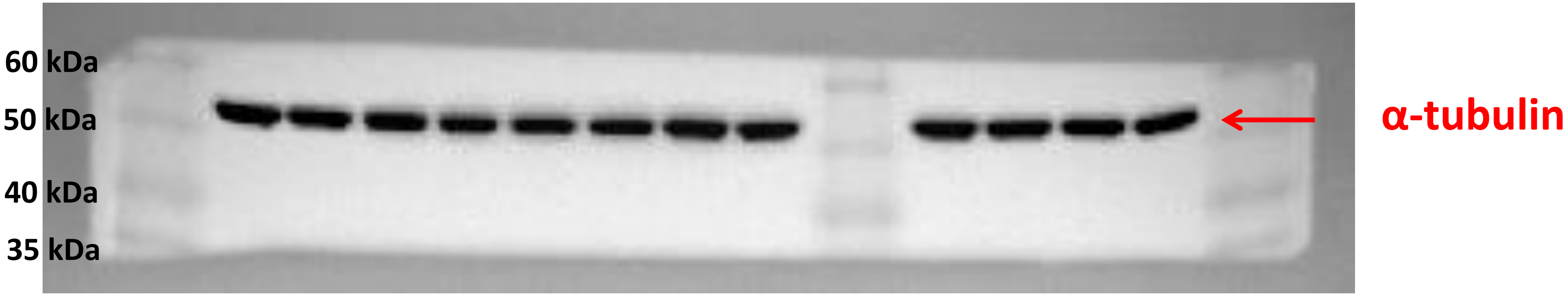

HT29 (N=3)

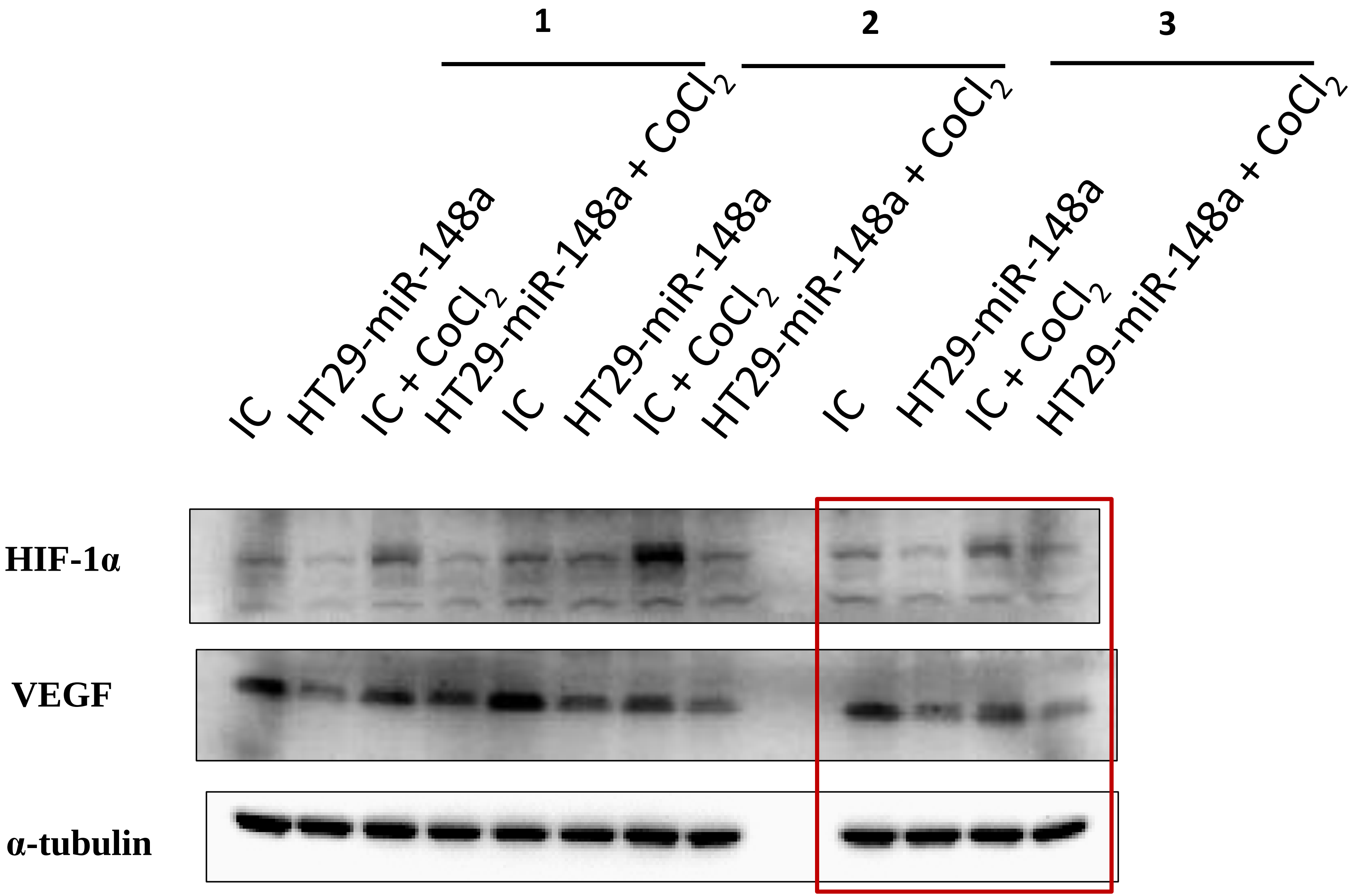
1
2
3
IC
HT29-miR-148a
IC + $CoCl_2$
HT29-miR-148a + $CoCl_2$
IC
HT29-miR-148a
IC + $CoCl_2$
HT29-miR-148a + $CoCl_2$
IC
HT29-miR-148a
IC + $CoCl_2$
HT29-miR-148a + $CoCl_2$
HIF-1α
VEGF
α-tubulin

Supplementary Figure 6

A HCT116 (N=3)

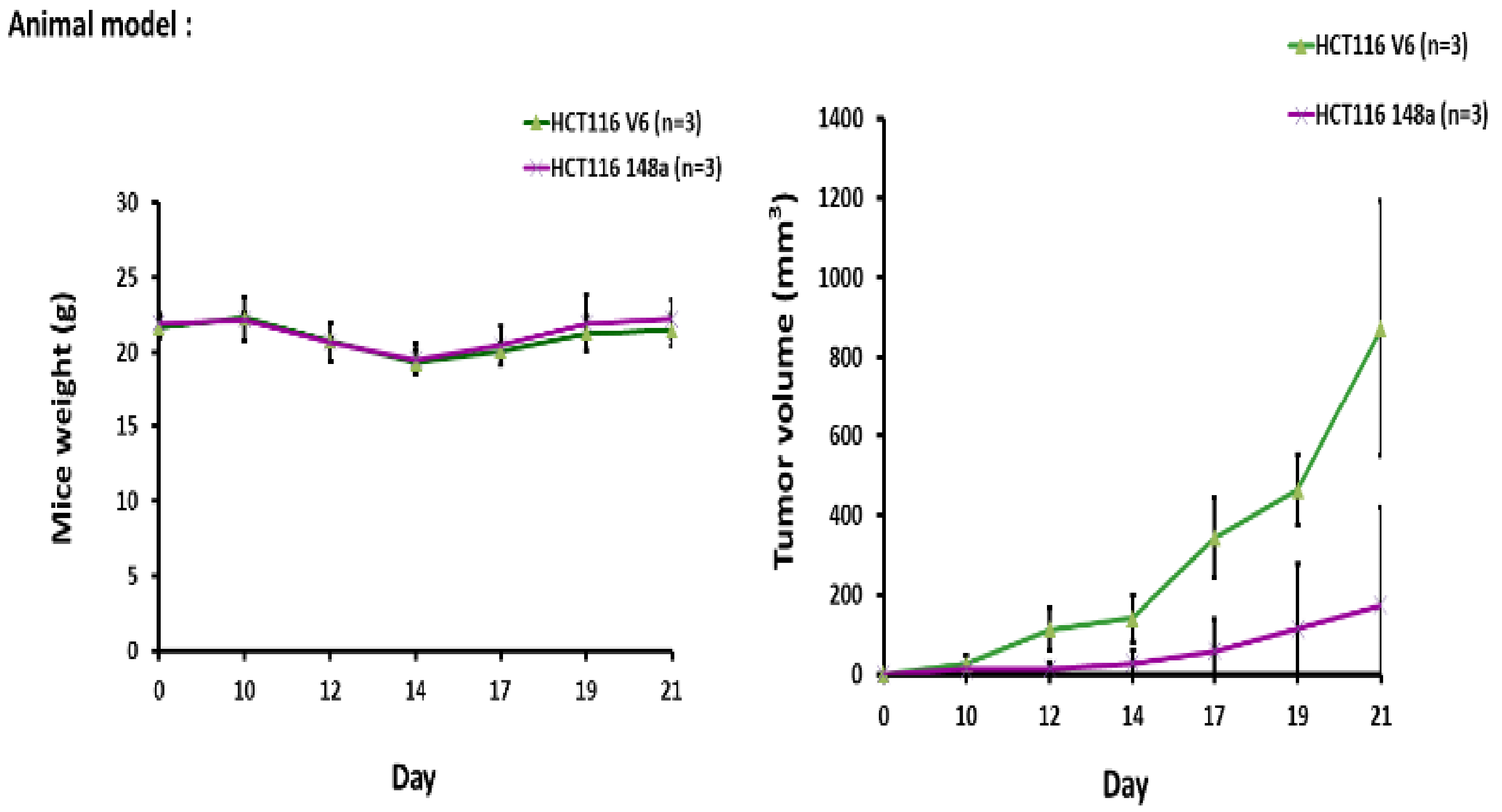

Tumor burden :

Tumor lumps were smaller in miR-148a group

HCT116 V6

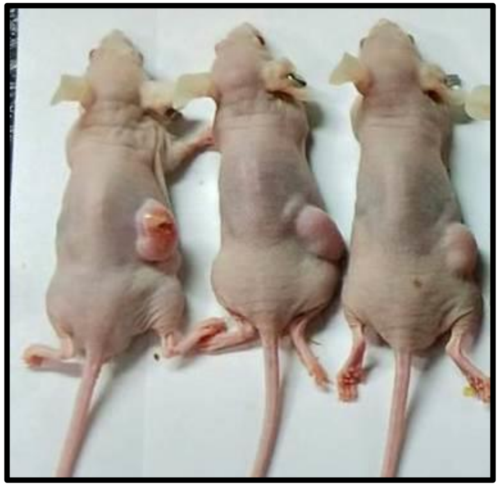

HCT116 148a

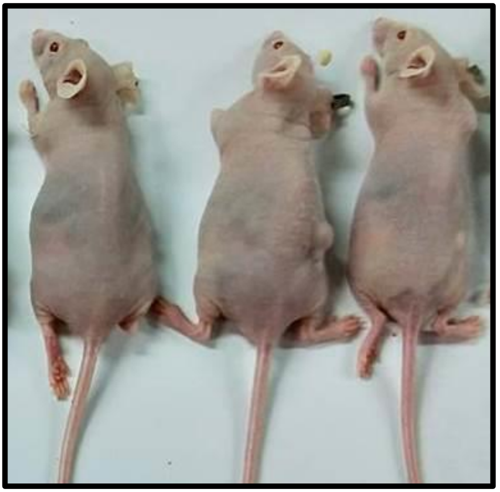

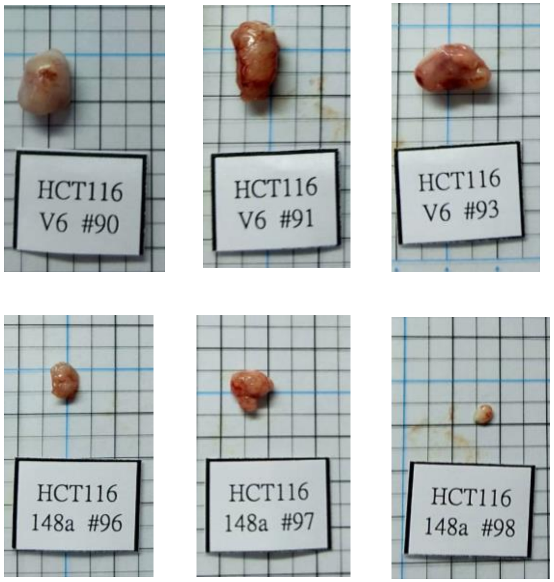

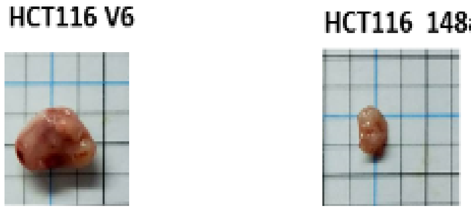

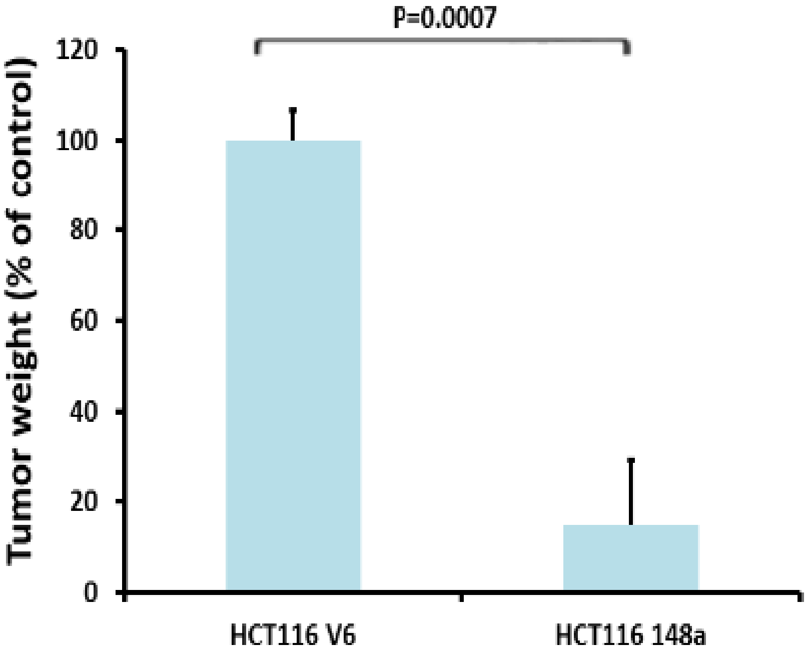

B HT 29 (N=3)

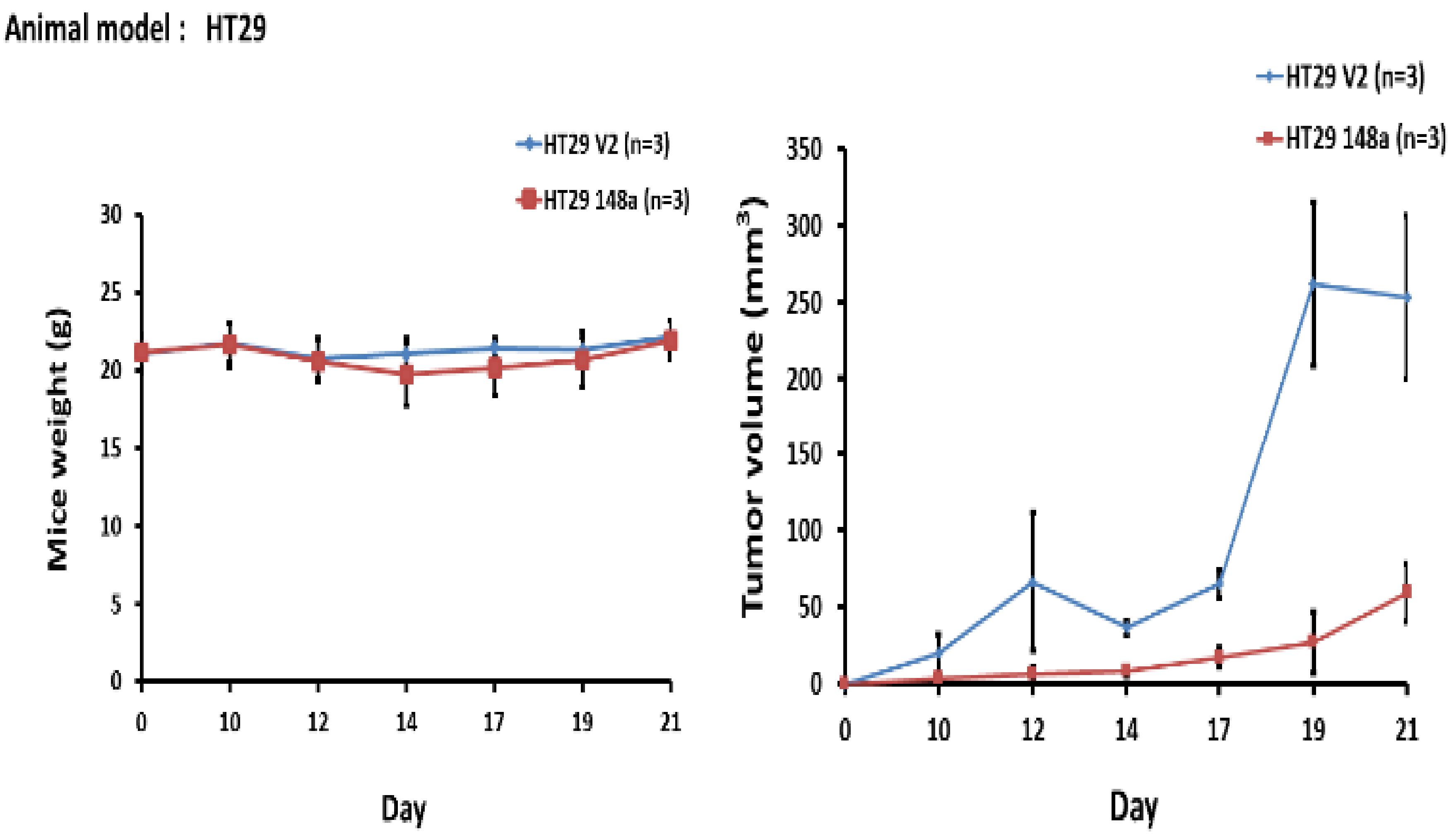

Animal model : HT29
HT29 V2 (n=3)
HT29 148a (n=3)
Mice weight (g)
30
25
20
15
10
5
0
0 10 12 14 17 19 21
Day
HT29 V2 (n=3)
HT29 148a (n=3)
Tumor volume (mm^3)
350
300
250
200
150
100
50
0
0 10 12 14 17 19 21
Day

Tumor burden :

Tumor lumps were smaller in miR-148a group

HT29 V2

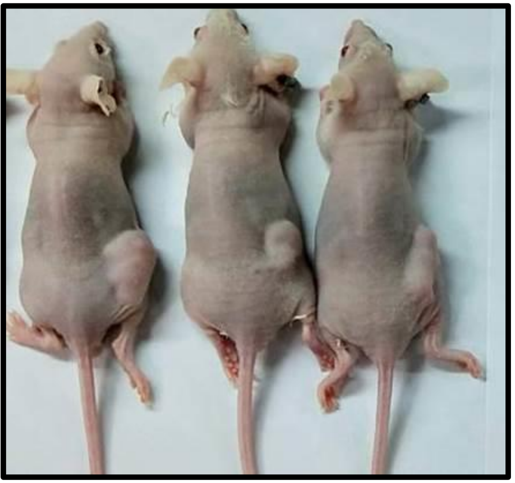

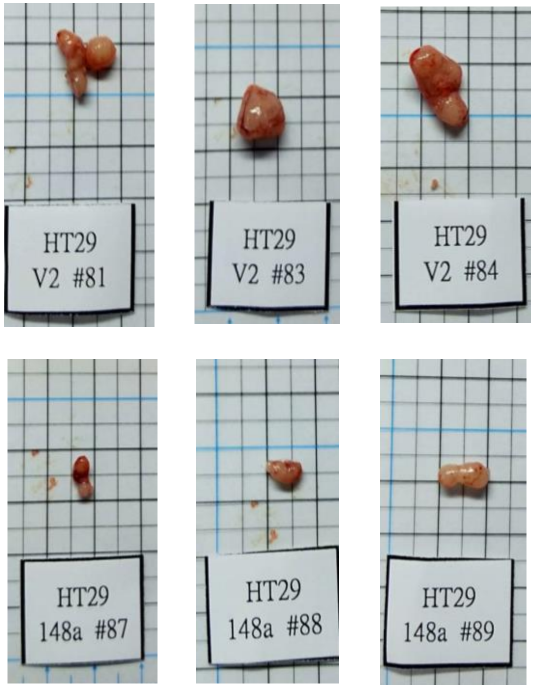

HT29 148a

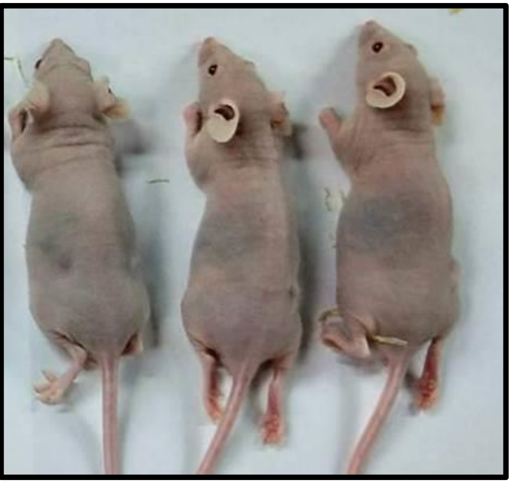

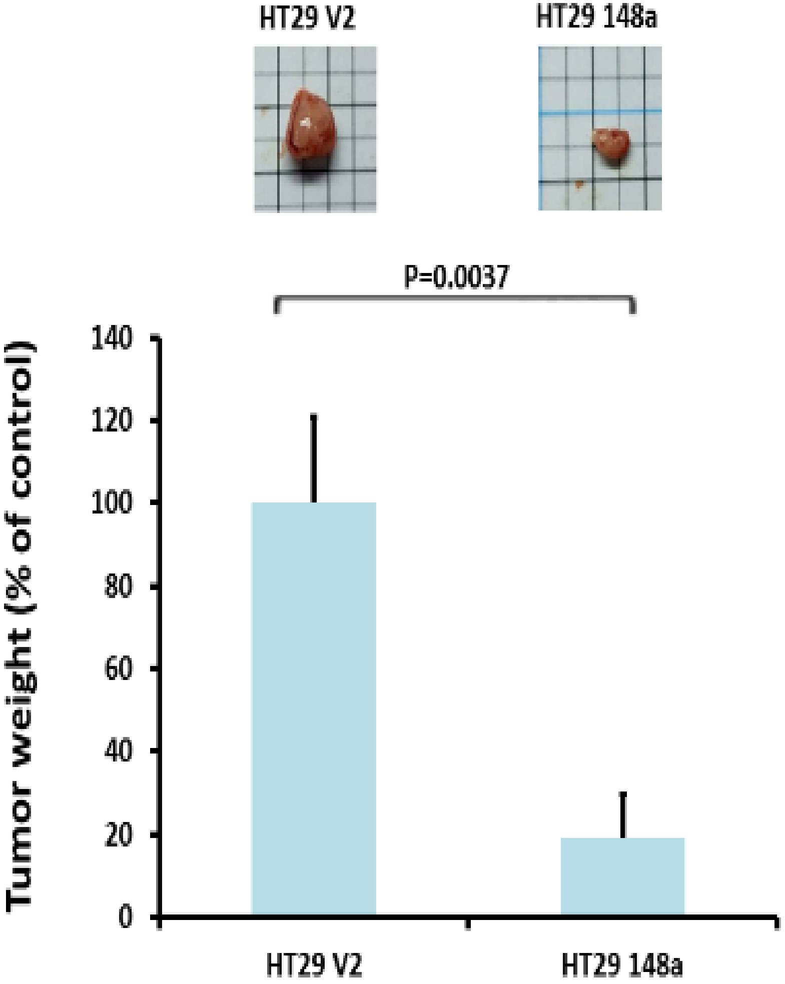

Supplement: Supplementary file 1 [file JCMM-23-3572-s001.pdf]
