# Supplementary material for: miR‐148a inhibits early relapsed colorectal cancers and the secretion of VEGF by indirectly targeting HIF‐1α under non‐hypoxia/hypoxia conditions
Source: J Cell Mol Med. 2019 Mar 4;23(5):3572–82. doi: 10.1111/jcmm.14257 (PMC6484316; doi:10.1111/jcmm.14257)
Supplement: Supplementary file 2 [file JCMM-23-3572-s002.docx]

**FIGURE S1** *miR-148a* was transfected into 5 cell lines. A, *miR-148a* was successfully transfected to HCT116 (7-fold) and HT29 (140-fold). B, *miR-148a* was not significantly transfected into SW480 (2.3-fold); SW620 (1.75-fold); and not successfully performed in Caco-2.

**FIGURE S2** *miR-148a* doesn’t directly bind 3’-UTR of HIF-1α by Luciferase assay. NC vector indicates empty vector. A, In the HCT116-*miR-148a* cell line, there is not significantly different between the NC vector and HIF-1α 3’UTR vector (*P* = 0.71). B, In HT29-*miR-148a* cell line, it is also not significant difference between NC vector and HIF-1α 3’UTR vector (*P* = 0.838).

**FIGURE S3** Overexpression of *miR-148a* in HCT116 and HT29 cell lines inhibits the expression of HIF-1α and VEGF in non-hypoxic and hypoxic culture conditions created by use of CoCl_2_. IC indicates HCT116 or HT29 cell line. A, In HCT116 cell line, the expressions of HIF-1α and VEGF were inhibited by *miR-148a* in non-hypoxic/hypoxic conditions (*P* = 0.036 and 0.036, 0.006 and 0.045; respectively). B, In HT29 cell line, the expressions of HIF-1α and VEGF were also inhibited by *miR-148a* in non-hypoxic/hypoxic conditions (*P* = 0.026 and 0.021, 0.002 and 0.041; respectively).

**FIGURE S4** HUVEC tube formation assay. *miR-148a*-medicated inhibition of angiogenesis was measured and statistically significant between control colon cancer cell line and *miR-148a*-expressing colon cancer cell lines. A, In HCT116 cell (*P* = 0.02). B, In HT29 cell (*P* = 0.03).

**FIGURE S5** The full length blots/gels of HIF-1α and VEGF. A, In HCT116 cell line, the molecular weight of HIF-1α, VEGF and α-tubulin was 100 kDa, 25 kDa, and 50 kDa. B, In the HT29 cell line, the molecular weights of HIF-1α, VEGF, and α-tubulin were also 100, 25, and 50 kDa.

**FIGURE S6** Effect of *miR-148a* on tumor growth in an animal model. A, In the HCT116 cell line, tumor lumps were smaller and tumor growth was slower in *OmiR-148a* cells (*P* = 0.0007). B, In the HT29 cell line, tumor lumps were smaller and tumor growth was slower in *OmiR-148a* cells (*P* = 0.0037).
